# Bolaamphiphilic Bis-Dehydropeptide Hydrogels as Potential Drug Release Systems

**DOI:** 10.3390/gels7020052

**Published:** 2021-04-29

**Authors:** Carolina Amorim, Sérgio R. S. Veloso, Elisabete M. S. Castanheira, Loic Hilliou, Renato B. Pereira, David M. Pereira, José A. Martins, Peter J. Jervis, Paula M. T. Ferreira

**Affiliations:** 1 Center of Chemistry, University of Minho, 4710-057 Braga, Portugal; carolinaamorim753@gmail.com (C.A.); jmartins@quimica.uminho.pt (J.A.M.); 2 Center of Physics, University of Minho, 4710-057 Braga, Portugal; sergioveloso96@gmail.com (S.R.S.V.); ecoutinho@fisica.uminho.pt (E.M.S.C.); 3 Institute for Polymers and Composites, University of Minho, 4800-058 Guimarães, Portugal; loic@dep.uminho.pt; 4 REQUIMTE/LAQV, Laboratório de Farmacognosia, Departamento de Química, Faculdade de Farmácia, Universidade do Porto, R. Jorge Viterbo Ferreira, n 228, 4050-313 Porto, Portugal; rjpereira@ff.up.pt (R.B.P.); dpereira@ff.up.pt (D.M.P.)

**Keywords:** bolaamphiphiles, dehydropeptide, self-assembly, supramolecular hydrogels, drug delivery

## Abstract

The self-assembly of nanometric structures from molecular building blocks is an effective way to make new functional materials for biological and technological applications. In this work, four symmetrical bolaamphiphiles based on dehydrodipeptides (phenylalanyldehydrophenylalanine and tyrosyldehydrophenylalanine) linked through phenyl or naphthyl linkers (terephthalic acid and 2,6-naphthalenedicarboxylic acid) were prepared, and their self-assembly properties were studied. The results showed that all compounds, with the exception of the bolaamphiphile of tyrosyldehydrophenylalanine and 2,6-naphthalene dicarboxylic acid, gave self-standing hydrogels with critical gelation concentrations of 0.3 wt % and 0.4 wt %, using a pH trigger. The self-assembly of these hydrogelators was investigated using STEM microscopy, which revealed a network of entangled fibers. According to rheology, the dehydrodipeptide bolaamphiphilic hydrogelators are viscoelastic materials with an elastic modulus G′ that falls in the range of native tissue (0.37 kPa brain–4.5 kPa cartilage). In viability and proliferation studies, it was found that these compounds were non-toxic toward the human keratinocyte cell line, HaCaT. In sustained release assays, we studied the effects of the charge present on model drug compounds on the rate of cargo release from the hydrogel networks. Methylene blue (MB), methyl orange (MO), and ciprofloxacin were chosen as cationic, anionic, and overall neutral cargo, respectively. These studies have shown that the hydrogels provide a sustained release of methyl orange and ciprofloxacin, while methylene blue is retained by the hydrogel network.

## 1. Introduction

Supramolecular hydrogels have attracted considerable attention due to their potential applications as promising biomaterials for many biotechnological and biomedical applications, such as drug delivery [1,2], cell culture [3], tissue engineering [4], wound healing [5,6], and many others. Upon receiving an external trigger, short peptides capped with aromatic groups on the *N*-terminus can often self-assemble into three-dimensional (3D) networks, forming fibers that can trap water molecules, providing biocompatible and biodegradable supramolecular hydrogels [7]. The process of self-assembly occurs by means of non-covalent interactions such as hydrogen bonding, aromatic π–π stacking, hydrophobic, ionic, and Van der Waals interactions to form long fibrous nanostructures. Several methods have been reported for triggering gelation such as pH change, temperature change, and enzymatic modification of the gelator [5,6,8]. For efficient hydrogelation, it is necessary to have a balance of hydrophobic and hydrophilic groups in order to establish the intermolecular interactions required for fiber network formation and immobilization of water molecules [9].

Numerous examples of supramolecular peptide hydrogels exist in the literature, particularly those consisting of di- and tripeptides. Typically, the *N*-terminus of the di- or tripeptide is functionalized with a large aromatic group, such as fluorenylmethoxycarbonyl (Fmoc) [10], naphthalene (Nap) [11], naproxen (Npx) [12,13,14], or other aromatic group, while the *C*-terminus is usually unprotected as the free carboxylic acid. Despite the high biocompatibility and bioavailability of these supramolecular hydrogels, their susceptibility to enzymatic hydrolysis is a major limitation for the use of peptide-based pharmaceuticals in general, including as hydrogels. One method for increasing the proteolytic stability of these peptides is to employ a peptidomimetic strategy. In such a strategy, non-proteinogenic amino acids, such as D-amino acids, β-amino acids, or dehydroamino acids, are incorporated into the peptide chain of hydrogelators, which are more stable to enzymatic hydrolysis [12,13].

**Figure 1:**
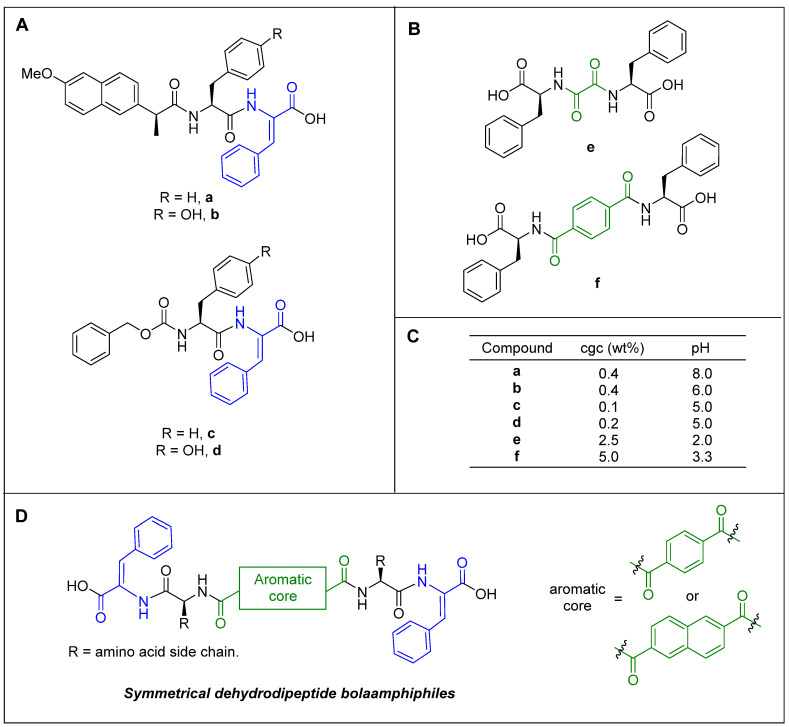
(**A**) Our previously studied naproxen- and carboxybenzyl-capped dehydropeptide hydrogelators. (**B**) Examples of bolaamphiphilic hydrogelators in the literature. (**C**) Summary of hydrogel properties. (**D**) Combining the structures of *N*-capped dehydrohydropeptide gelators and bolaamphiphilic gelators into a single molecule.

Our research group, and others, have reported new dehydropeptides capable of forming hydrogels. The presence of a dehydroamino acid residue imparts increased proteolytic resistance into the hydrogels, as well as different physical properties arising from the decreased structural flexibility of the dehydropeptide chain [13,14,15,16]. In our laboratories, several dehydrodipeptides capped on the *N*-terminus with naproxen (e.g., Npx-L-Phe-*Z*-ΔPhe-OH and Npx-L-Trp-*Z*-ΔPhe-OH) were prepared and tested as hydrogelators, providing hydrogels with critical gelation concentrations (CGCs) of between 0.4 and 0.6 wt % and a gelation pH of between 5 and 8. These materials showed promise as controlled drug delivery systems, in release studies using anti-cancer compounds such as curcumin and doxorubicin [12,13]. More recently, we found that compounds containing a structurally simpler carboxybenzyl (Cbz) group attached to the *N*-terminus of dehydrodipeptides (i.e., Cbz-L-Phe-*Z*-ΔPhe-OH and Cbz-L-Tyr-*Z*-ΔPhe-OH) are capable of forming hydrogels with very low CGCs (0.1%) and therefore represent a new minimum gelator module for this class of gelator, at least under the gelation conditions tested (Figure 1A) [17].

In an alternative strategy for accessing hydrogels with novel properties, several research groups have investigated homodimeric hydrogelators, possessing bolaamphiphilic structures. Bolaamphiphilic gelators are composed of two polar heads at both ends of a central hydrophobic chain, which effectively doubles the amount of supramolecular interactions contained within the gel structure whilst maintaining essentially the same overall polarity of the corresponding monomer. These hydrogelators have shown interesting properties compared with their monomeric counterparts [18,19]. Although these bolaamphiphilic hydrogelators are necessarily higher in molecular weight, the symmetrical structure can still be rapidly accessed by two-directional synthesis. Therefore, the number of synthetic steps remains the same as for obtaining the corresponding monomers. Some examples of bolaamphiphilic hydrogelators are shown in Figure 1B [20,21]. When gelation is triggered by a pH change, the gelation process is potentially a more complicated process, as gelation can potentially occur following the protonation of one or two of the carboxylate groups. Therefore, the optimal gelation pH may be more difficult to predict, but it is generally lower than that of monomeric versions [22]. The differences in gelation pHs are highlighted in Figure 1C.

In this work, we were keen to adapt our previous dehydrodipeptide hydrogelators into bolaamphiphilic versions in order to improve the physical properties (Figure 1D). Combining these two structures (bolaamphiphiles and dehydropeptides) into a single molecule would increase the number of interactions in the gel structure whilst increasing the stability to proteolytic degradation and reducing the molecular flexibility of the molecules. Furthermore, the benefits of a short synthetic sequence would be retained. The dimeric nature of a bolaamphiphilic hydrogel would also be expected to provide a higher density of interactions within the gel structure than longer non-symmetrical peptides of equivalent molecular weight.

**Figure 2:**
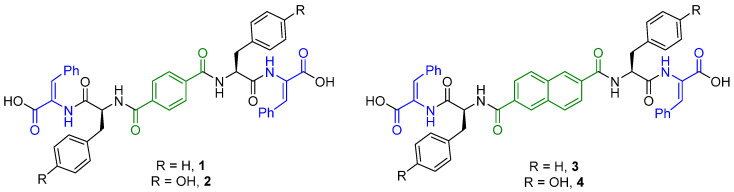
Chemical structures of the studied dehydrodipeptide derivatives **1**, **2**, **3** and **4**.

To this end, we now report the synthesis and characterization of new bolaamphiphilic bis-dehydropeptides **1**–**4**, where the *N*-terminus of the dehydrodipeptide was connected to both ends of a bifunctional central aromatic moiety, namely 1,4-benzenedicarboxylic acid or naphthalene-2,6-dicarboxylic acid, to create homodimeric amphiphilic molecules (Figure 2). These compounds were evaluated as novel hydrogelators, and the properties were compared with our previously reported and similar monomeric versions of these hydrogelators. The self-assembly of these hydrogels was investigated by rheometry and STEM microscopy. The biocompatibility of these compounds was assessed by cytotoxicity assays. In sustained release assays, we studied the effects of the charge present on the model drug compound on the rate of cargo release from the hydrogel networks. Methylene blue (MB), methyl orange (MO), and ciprofloxacin were chosen as cationic, anionic, and overall neutral cargo, respectively.

## 2. Results and Discussion

### 2.1. Synthesis

**Scheme 1:**
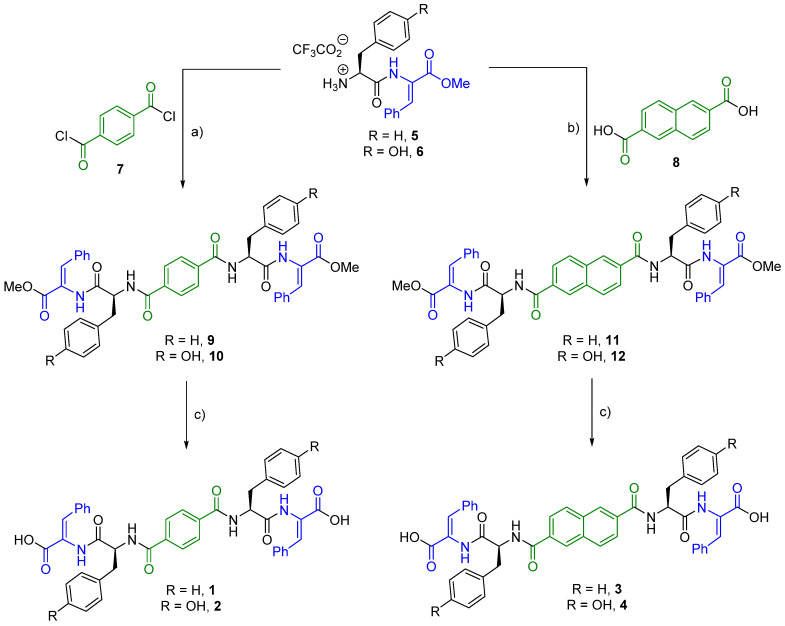
Synthesis of compounds **1**–**4**. (a) Et_3_N dry THF, N_2_ atm, reflux, 80 °C; (b) MeCN or DMF, Et_3_N, HBTU; (c) 1. NaOH, 1,4-dioxane, 2. KHSO_4_.

The synthesis of bolaamphiphilic bis-dehydropeptides **1**–**4** is shown in Scheme 1. The intermediates H-L-Phe-*Z*-ΔPhe-OMe.TFA **5** and H-L-Tyr-*Z*-ΔPhe-OMe.TFA **6** were synthesized by a known route [13]. The reagents terephthaloyl chloride (**7**) and naphthalene-2,6-dicarboxylic acid (**8**) were employed to install the central aromatic portion. Thus, dimeric dehydropeptides **1** and **2** were prepared in good yields by a double nucleophilic substitution of **5** and **6** onto the diacyl chloride **7**, whereas dimeric dehydropeptides **3** and **4** were prepared in high yields by a double amide coupling protocol of **5** and **6** onto diacid **8**, employing HBTU. Ester hydrolyses using NaOH converted compounds **9**–**12** into the target diacids **1**–**4** in high yields. 

### 2.2. Gelation Study

Bola-dehydrodipeptides **1**–**4** showed limited solubility in buffer solutions in the physiological pH range (6–8). Nonetheless, dimeric dehydropeptides **1**–**4** could be dissolved in water upon pH adjustment to pH 10–11, which was achieved by addition of NaOH solution (1 M). Gel formation was triggered by a slow pH drop. The pH change was obtained by the aqueous hydrolysis of added D-glucono-δ-lactone (GdL) to D-gluconic acid. In these conditions, compounds **1**–**3** gave self-standing hydrogels (Figure S1 in Supplementary Information). Bola-dehydrodipeptide **4** failed to give hydrogels using several gelification triggers (pH, temperature, and solvent exchange) and different concentrations (0.3–0.8 wt %). The CGCs of hydrogels **1**–**3** were qualitatively assessed by varying the peptide concentrations and conducting vial inversion tests. The hydrogels prepared with the compound **1**–**3** exhibit relatively low CGCs (0.3–0.4 wt %; 3–4 mg/mL) (Table 1).

**Figure 3:**
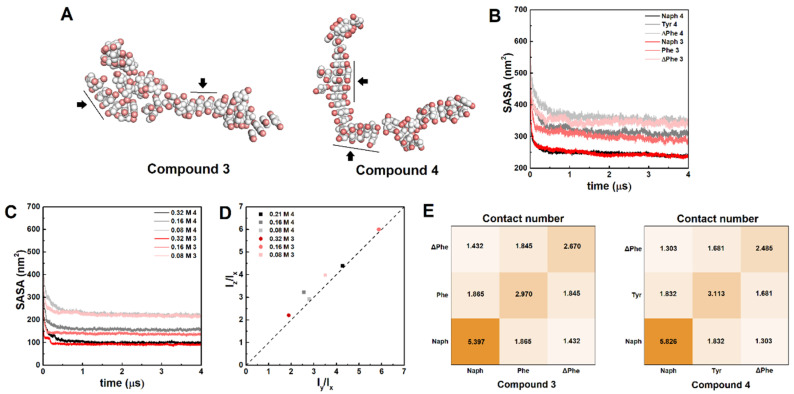
(**A**) Snapshot of the largest cluster resulting from the self-assembly of compound **3** and compound **4**, only depicting the 2,6-naphthalenedicarboxyl block. The arrows indicate stacking of the naphthalene moieties. Time evolution of the SASA for the (**B**) peptide residues and (**C**) aggregates in simulations of different concentration. (**D**) Moments of inertia of the clusters in the final snapshot for both compounds **3** and **4** in different concentrations, which for a one-dimensional aggregate: I_x_ << I_y_ ≈ I_z_. (**E**) Average contact number between different pairs of the compounds calculated from the last 2 µs of simulation.

In order to understand the different behavior of compound **4** and explore the self-assembly mechanisms of compounds **3** and **4**, coarse-grained molecular dynamics studies were carried out [23,24,25,26]. The assembly of compounds **3** and **4** at different concentrations was compared to evaluate the influence of a tyrosine residue instead of a phenylalanine close to the naphthalene moiety. Notably, both compounds assembled in less than 100 ns “effective time” at large concentrations (Figures S2 and S3 in Supplementary Information) and evolved toward long fibrils. However, the more hydrophobic compound **3** (cLogP = 6.69) formed thicker fibrils than compound **4** (cLogP = 5.55) in the simulation time. Closer inspection (Figure 3A) of the final self-assembled structures reveals a stacking of naphthalene moieties leading to the thin fibrils of compound **4**, while compound **3** displays smaller fibrils of stacked naphthalene moieties growing from thicker regions that exhibit randomly oriented naphthalene rings stabilized by the phenyl rings. The higher degree of aggregation of compound **3** is also translated in a larger empirical aggregation propensity score (Table S1 in Supplementary Information). Nonetheless, both compounds attain values (AP > 3) that fall in the range of peptides known to self-assemble into fibers and other structures [24,25,27].

Concerning the solvent accessible surface area (SASA), the exposed fraction of the tyrosine residue tends to increase for larger concentrations of compound **4** (Table S1 in Supplementary Information), and its presence leads to a more hydrated fibril without affecting the solvation of the naphthalene moiety (Figure 3B). Consequently, the self-assembly of the more hydrophobic compound **3** is characterized by a steeper SASA decrease than compound **4** (Figure 3C).

In the assessed concentration range, both compounds display a different behavior (Figure 3D). The increase of compound **4** concentration favors fibril elongation in two dimensions, while increasing compound **3** concentration led to three-dimensional fibril growth. Furthermore, the higher hydration of compound **4** fiber seems to lead to a reduction of contact between the dehydrophenylalanine residues when compared with compound **3**, while the interaction between the residues of naphthalene, involved in the formation of the fibril hydrophobic core, was enhanced as suggested by the larger average contact numbers (Figure 3E).

### 2.3. STEM Studies

**Figure 4:**
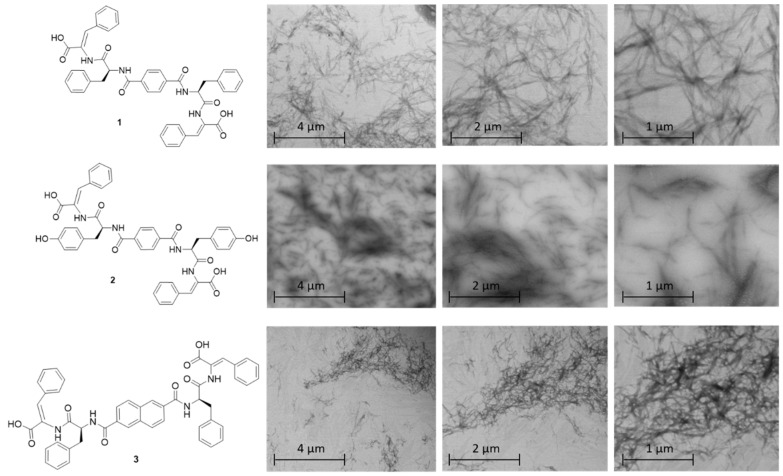
Comparison of scanning transmission electron microscopy (STEM) images of hydrogels **1** (top), **2** (center), and **3** at 0.4 wt %, at different magnifications (4, 2, and 1 μm).

The micro- and nanostructures of the new hydrogels were studied using scanning transmission electron microscopy (STEM). The STEM images of hydrogels obtained from compound **1**–**3** are shown in Figure 4. Compounds **1**–**3** display an entangled fibril structure that is the result of peptide self-assembly. In general, the fibers formed by compound **1** (Figure 4) display a thickness of around 35–47 nm, and the length is approximately 0.46–0.88 μm. Dehydropeptide **2** (Figure 4) shows fibers with diameters around 35–47 nm and lengths between 0.6 and 1.8 μm. Finally, compound **3** (Figure 4) shows fibers with a thickness around 17–27 nm and lengths between 0.3 and 0.8 μm.

The STEM measurements reported for the dehydrodipeptides phenylalanyldehydrophenylalanine and tyrosyldehydrophenylalanine *N*-capped with the carboxybenzyl group are similar to those described for bolaamphiphiles **1**–**3** (Figure S4 in Supplementary Information) [17]. The average width reported for the fibers of these hydrogels is 26.1 nm. The Cbz-capped hydrogels show more connectivity than bolaamphiphiles **1**–**3**, which possess bulkier and more disconnected structures.

### 2.4. Circular Dichroism (CD) Spectroscopy

**Figure 5:**
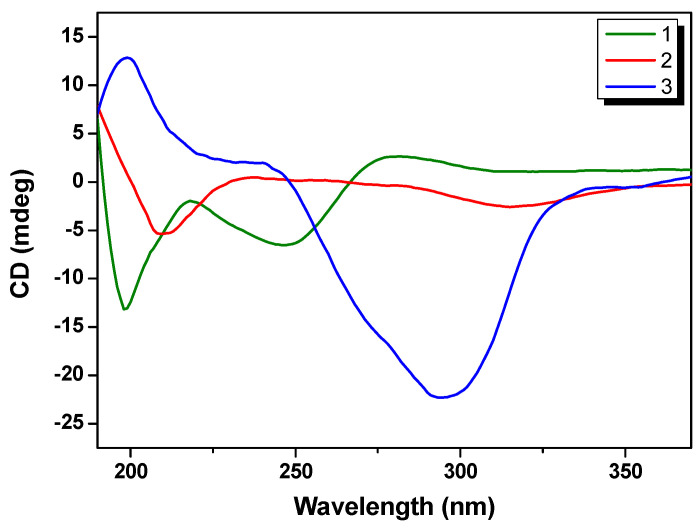
CD spectra of diluted aqueous solutions of compounds **1**, **2**, and **3**.

The CD spectra of hydrogelators **1**, **2**, and **3** are presented in Figure 5. To avoid the light-scattering effects that can occur in turbid gels, CD spectra were acquired with concentrations of hydrogelator solutions lower than the CGC. In the preparation of the samples to be analyzed by CD, the glucono-δ-lactone was added to the hydrogelator solutions to simulate the experimental conditions of the gel formation. The CD spectrum of compound **1** exhibits negative bands around 200 and 250 nm and a positive band at 220 nm (Figure 5, green line). Based on the shape of its spectrum, compound **1** seems to suggest a contribution of β-sheets and random coil. The CD spectrum of compound **2** shows a negative band at 220 nm and a negative broad peak around 320 nm (Figure 5, red line), which showed evidence of aggregating into a random coil. Finally, the CD spectrum of compound **3** shows two positive bands at 200 nm and 250 nm and a large negative peak around 290 nm (Figure 5, blue line), reflecting the importance that naphthalene interactions have in self-assembly.

### 2.5. Rheology

Rheology provides structural information about the type, density, and strength of the networks responsible for hydrogelation. It is common to investigate the mechanical properties of hydrogels in terms of the Young’s modulus, which is a measure of stiffness (geometry-independent). Native tissues and organs (e.g., skin, pancreas, spleen, glands, and muscles) possess a Young’s modulus in the range of 0.1 kPa (brain) to 1 MPa (cartilage), which is suited to the required function of the tissue in question. In tissue engineering applications, the hydrogel stiffness should closely match the proposed extracellular matrix (ECM) and be of sufficient stiffness to sustain cell growth for the required amount of time. Alternatively, in drug delivery applications, the gel stiffness should be sufficient to allow the gel structure to be maintained for the duration of the drug release time period [28].

The gelation kinetics of compounds **1** and **2** (Figure S5 in Supplementary Information) show that G′ is significantly larger than G″ after 2 h, which indicates that the hydrogels formed relatively quickly, within the same time scale of gel formation for carboxybenzyl-protected dehydropeptides Cbz-L-Phe-Z-ΔPhe-OH and Cbz-L-Tyr-Z-ΔPhe-OH [17], but faster than some Fmoc-dipeptides [29]. Bolaamphiphile **1** shows an initial time lag of 2300 s before the significant rise in G′ and the final slower equilibration step, while for compound **2**, this time is longer (4600 s). The scattering of data recorded with compound ***3*** at early time points does not allow a quantitative assessment of the gel kinetics, which seems complete after 4 h (Figure S5 in Supplementary Information). This is due to the rather small deformation used during this test to allow gel formation without disturbing the build-up of the network structure. In contrast to this, see the data for compounds **1** and **2**. A two-step model is often used to describe the nucleation (with rate constant kn) and growth (with rate constant k) of aggregates of peptides [30]:

(1) $$f\left(t\right)\;=\;G0\;+\;dG\frac{\rho \left(e^{\left(1+\rho \right)kt}\;-\;1\right)}{1\;+\;\rho e^{\left(1+\rho \right)kt}}$$

where $\rho \;=\;\frac{k_{n}}{k}$, *G*0 is the initial storage modulus of the aggregate-free solution, and *dG* is the increment in the storage modulus during the kinetics.

**Figure 6:**
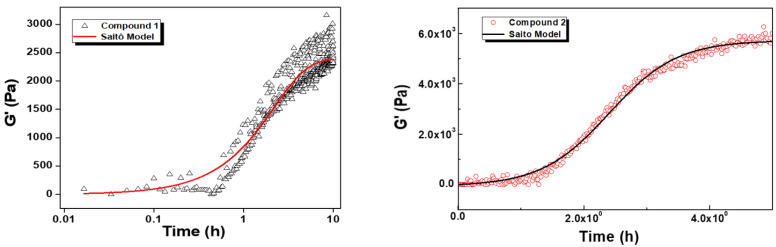
Time dependence of the storage modulus G′ (symbols) recorded during the gelling of compounds **1** and **2**, fitted with Equation (1)–line.

A fit of Equation (1) to the gel kinetics of compound **2** is presented in Figure 6, which shows that the model successfully reproduces the rheological data if one accepts that the parameter G0 has no meaning, since the aggregate-free solution has no measurable elasticity. A less successful fit was achieved for the kinetics for compound **1** (Figure 6). This is explained by the scatter in the data and by the fact that gelling is not fully completed after 10 h. However, the errors in the values computed for the rate constants (Table 2) are small enough to offer a quantitative comparison between the gelling kinetics of the two compounds.

A comparison of the parameters of Equation (1), computed from fitting the rheological data of compounds **1** and **2**, is shown in Table 2. The latter shows that nucleation and growth of peptide aggregates occurs on the same time scale for compound **1**, whereas nucleation is much slower for compound **2**. The values computed for the rate constants of compound **2** are consistent with the values reported recently from turbidity experiments of a hydrogel based on a dehydrodipeptide *N*-protected with a carboxybenzyl group [17].

After reaching the structural equilibrium established by the reading of constant shear moduli G′ and G″ with time, a frequency-sweep from 100 down to 0.1 Hz was performed with a strain of 0.01% (Figure S6 in Supplementary Information). For hydrogels obtained from compounds **1**, **2**, and **3**, G′ is essentially constant over the frequency domain tested, and it is 10 times larger than G″. Overall, all mechanical spectra are qualitatively similar and reminiscent of the rheological signature of gels, as G′ is higher than G″ for hydrogels of compounds **1**–**3** (Table 3). ′

**Figure 7:**
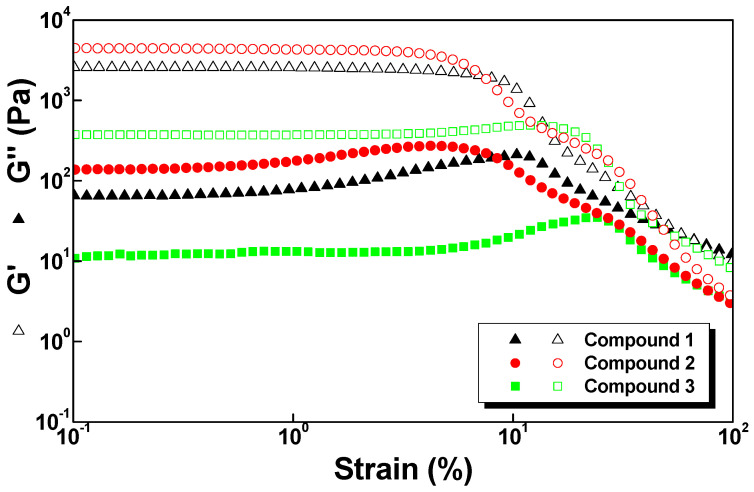
Strain dependence of the shear elastic G′ (empty symbols) and loss G″ (filled symbols) moduli for compounds **1**, **2**, and **3**.

The thermal stability of gels formed by compounds **1**–**3** (Figure S7 in Supplementary Information) was evaluated, and whereas the strain used with compound **3** was too small to detect any thermal variation in G′, the gel made from compound **1** is not thermally sensitive, and the gel made from compound **2** shows a thermal hysteresis. Interestingly, this compound, which was found to exhibit coil conformers under gelling conditions, forms a network that exhibits some entropic elasticity (see the increase in G′ with increasing temperature in Figure S7 in Supplementary Information). Overall, the results show that these gels can sustain body temperature with no significant structural changes. After the thermal cycle, gels were submitted to a strain sweep, where the frequency was maintained at 1 Hz. The hydrogels of compounds **2** and **1** break up more easily than hydrogel of compound **3** (Figure 7). These results suggest that the thickness of the nanofibers does not have a direct correlation with the strength of the hydrogel. The hydrogel of compound **3** has thinner fibrils but shows an increase in G′ before breaking (strain hardening) in contrast to hydrogels from compounds **1** and **2**, which show a continuous drop of G′ before breaking. This suggests a different structure for the hydrogel of bolaamphiphile **3**, which is reminiscent of filamentous gels possessing a smaller elastic modulus (see *G0*) but showing elastic strengthening under strain. In summary, and connecting the rheological data to the structural information inferred from both STEM and CD, coil conformers of compound **2** rapidly self-assemble to build a rubber-like (entropic) network, which brings most of the gel elasticity. This is in contrast to the elastically weaker gels of compound **3**, which are predominantly made from the networking of fibrils likely formed from beta conformers. Gels of compound **1** show intermediate elasticity, originating from the slower networking of a mixture of coil and beta sheet conformers.

### 2.6. Biocompatibility and Cytotoxicity Studies

**Figure 8:**
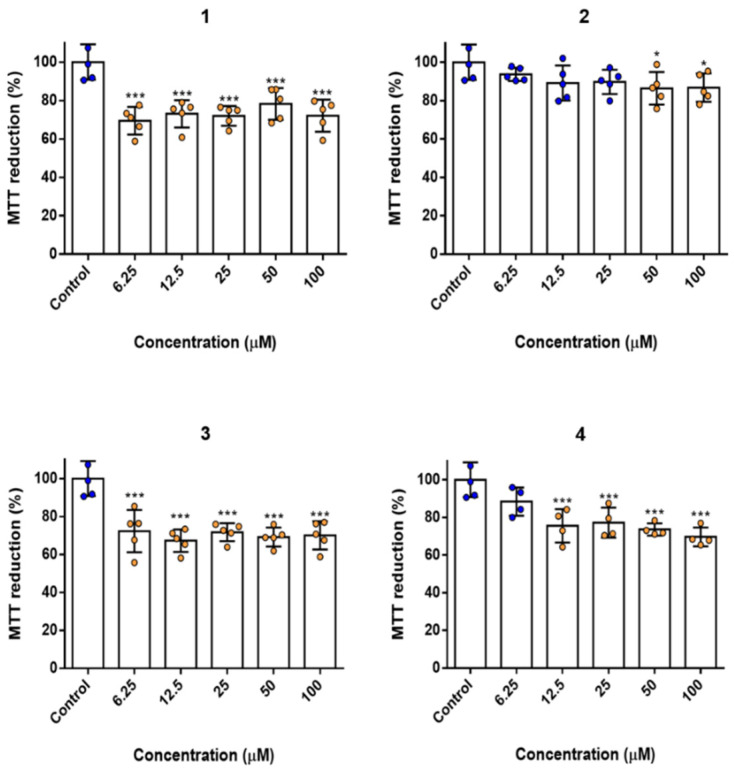
MTT reduction of HaCaT cells treated with compounds **1**, **2**, **3**, and **4** for 24 h, at the concentrations presented. * *p* < 0.05, *** *p* < 0.001.

The molecules under study were initially evaluated for their potential impact on the viability of human keratinocytes, namely the HaCaT cell line (Figure 8). Interestingly, compounds **1**, **3**, and **4** exhibited a similar behavior, with most concentrations tested eliciting a small apparent loss of cell viability, as assessed by mitochondrial activity, of around 20%, which was independent of concentration. On the other hand, compound **2** was mostly devoid of any effect, with the exception being the two highest concentrations, which show around 10% loss of mitochondrial activity.

In light of the results obtained for cell viability, we decided to further characterize the effects caused by the molecules under study. We have evaluated the effect of the molecules by assessing their impact in cell DNA and protein content as a strategy to identify potential changes in cell proliferation (Figure S8 in Supplementary Information). No statistically significant changes were found in any of the two parameters. These results show that the molecules under study have no identifiable impact in cell proliferation, despite having a small impact in mitochondrial activity. Relevantly, none of the molecules elicited loss of membrane integrity, which is a hallmark of necrosis (Figure S9 in Supplementary Information).

### 2.7. Drug Release Studies

**Figure 9:**
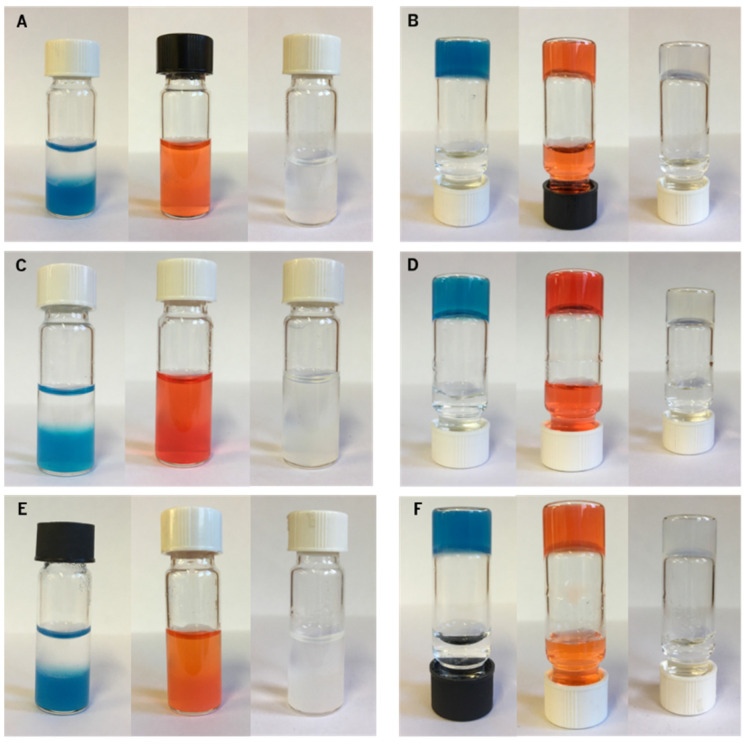
Representative images of hydrogels layered with water after a saturating release study (6 days). (**A**) Hydrogels of **1** loaded with methylene blue (left), methyl orange (center), and ciprofloxacin (right), that were layered with 1.5 mL of H_2_O. (**B**) Inverted vials from panel (**A**). (**C**) Hydrogels of **2** loaded with methylene blue (left), methyl orange (center), and ciprofloxacin (right), which were layered with 1.5 mL of H_2_O. (**D**) Inverted vials from panel (**C**). (**E**) Hydrogels of **3** loaded with methylene blue (left), methyl orange (center), and ciprofloxacin (right), which were layered with 1.5 mL of H_2_O. (**F**) Inverted vials from panel (**E**).

Hydrogels containing cationic, anionic, or zwitterionic model drugs were prepared using the same conditions described in Section 2.2 but with the water component (1 mL) being replaced by a methylene blue solution, methyl orange solution, or ciprofloxacin solution. In a modified version of the method described by Abraham et al. [1], water was carefully layered on top of the hydrogel surface. Then, the percentage release of model drug was recorded versus time, over 6 days, through UV absorption measurements or by HPLC and then subsequently fitted to a standard calibration curve (Figure S10 in Supplementary Information). The experiments were also assessed visually (Figure 9). In experiments involving hydrogels of **1**–**3** containing cationic methylene blue, the top layer stayed almost colorless and transparent over several days, suggesting that the dye is retained by the hydrogel network (Figure 9B,D,F left). These results were confirmed by UV absorption measurements (Table 4) (Figure S11 in Supplementary Information). In the studies involving the release of methyl orange, a clearly visible release of the anionic dye into the layered water solution was observed from the three hydrogels after several days (Figure 9B,D,F centre). The results for the release of methyl orange were also confirmed quantitatively by UV absorption measurements. Hydrogelators **1** and **3**, which contain a phenylalanine residue, release nearly 60% of the anionic methyl orange cargo, whereas hydrogel **2**, containing a tyrosine residue, released more than 90%. This result can be explained by the presence of the OH groups of the tyrosine residue, which may be deprotonated to a small extent in the gel state, providing additional repulsion of the anionic methyl orange (Table 4) (Figure S11 in Supplementary Information). Ciprofloxacin was included as an overall neutral cargo. In experiments involving hydrogels of compounds **1**–**3** containing ciprofloxacin, the amount of ciprofloxacin present in the above water layer was determined by HPLC at various time-points. Hydrogelators **1**, **2**, and **3** release 32%, 58%, and 20% of their ciprofloxacin cargo, respectively (Table 4) (Figure S11 in Supplementary Information). As observed for methyl orange, the hydrogel that releases the greatest amount of ciprofloxacin is the hydrogel obtained from compound **2** (Table 4, Figure S11 in Supplementary Information).

To quantitatively evaluate the drug release from hydrogels **1**–**3**, several mathematical models were tested to see which one fits the tested hydrogels. The Korsmeyer–Peppas model is the preferred model for polymeric systems. Korsmeyer described a simple relationship that described drug release from a polymeric system that includes both the diffusion and erosion of the polymer chain. The following equation describes the Korsmeyer–Peppas model [25,31]:

$$\frac{M_{t}}{M}\;=\;Kt^{n}$$

$M_{t}$: amount of cargo released at time t;

M: total amount of cargo used for the release study;

K: release rate constant incorporating structural and geometric characteristics of drug dosage form;

n: release exponent.

The determined parameters of this model (*K* and *n*) and the value of *R^2^* are presented in Table 5 (Figures S12 and S13 in Supplementary Information). These results showed that hydrogel **1** is faster (higher *K* value) and is associated more with a diffusion-controlled release mechanism (lower *n* value) than the other two hydrogels, relative to the release of ciprofloxacin. In the case of methyl orange, hydrogel **2** was shown to be the most effective hydrogel, as it presents the higher *K* and lower *n* value. As we can see in Table 5, the Korsmeyer–Peppas model exhibits good $R^{2}\;$values, which shows that this model is appropriate for the hydrogels under study.

## 3. Conclusions

In this work, we describe the synthesis, characterization, and gelation properties of four new bis-dehydropeptide bolaamphiphiles **1**–**4**, containing a phenylalanine or tyrosine residue connected to a dehydroamino acid residue at the *C*-terminus. The *N*-terminus of the dipeptide was connected to both ends to a benzene-1,4-dicarbonyl or a naphthalene-2,6-dicarbonyl. Three of the four compounds prepared behaved as efficient molecular hydrogelators, forming hydrogels with minimum gelation concentrations of 0.3–0.4 wt %. The rheology properties of hydrogels prepared were studied by oscillatory rheology, and the results showed a storage modulus (G′) significantly higher that their loss modulus (G″), which confirmed that they exhibit a viscoelastic behavior, which is characteristic of supramolecular hydrogels. STEM microscopy revealed that the self-assembled hydrogels display fibrillar structures. The biocompatibility of these compounds was assessed by cytotoxicity assays. These compounds were initially evaluated for their potential impact on the viability of human keratinocytes (HaCaT cell line) using MTT assays. We also evaluated their impact on cell proliferation by assessing their effect on cell DNA and protein content. The results show that the compounds synthetized have no identifiable impact on cell proliferation, despite having a small impact on mitochondrial activity.

Finally, in sustained release assays, we studied the effects of the charge present on model drug compounds on the rate of cargo release from the hydrogel networks, using cationic (methylene blue), anionic (methyl orange), and neutral cargo (ciprofloxacin). The results obtained showed that the hydrogels provide the retention of the methylene blue inside the hydrogel network, and a sustained release of methyl orange (60–90% release after 6 days) and ciprofloxacin (20–58% release after 6 days). In the experiments involving the methyl orange and ciprofloxacin, the hydrogel composed of compound 2, containing tyrosine residues, released more (90% and 58%, respectively) model drug compounds than hydrogels **1** and **3**, which contain phenylalanine residues. This can be explained by the presence of the OH groups of the tyrosine, providing an additional repulsion.

In summary, four new bolaamphiphilic bis-dehydrodipeptides **1**–**4** have been synthesized and characterized. Of these, compounds **1**–**3** were able to form hydrogels with low CGCs. The rheological profile of these hydrogels was close to that observed in biological tissues. Hydrogels **1**–**3** show promise as drug delivery vehicles, providing a sustained releases of model drug compounds over 6 days. The new compounds were shown to be biocompatible, presenting low toxicity to HaCaT mammalian cells at the highest concentrations tested (100 mM). There are many reports that demonstrate the proteolytic resistance of the dehydrodipeptide motif, and therefore, these hydrogelators are expected to be more enzymatically stable than corresponding structures containing only canonical amino acids [12,13,32,33,34,35]. Considering the rapid, inexpensive, and scalable synthesis of these hydrogelators, they should be of interest to the wider scientific community.

## 4. Materials and Methods

### 4.1. Synthesis

Compounds **1**–**4** were prepared by synthetic methodologies developed in our laboratory and fully characterized by ^1^H and ^13^C NMR spectroscopy (NMR) and High-Resolution Mass Spectrometry (HRM). Spectra were acquired on a Bruker Avance III 400 spectrometer, operating at 400.13 MHz and 100.62 MHz, for ^1^H and ^13^C NMR respectively. HRMS data were acquired from the mass spectrometry service of the University of Vigo, Spain.

#### 4.1.1. Compound **9**

To a stirred suspension of terephthaloyl chloride (**7**) (1.0 equiv., 0.25 mmol, 0.05 g) in dry THF (5 mL), Et_3_N (5.0 equiv., 1.25 mmol, 0.18 mL) and H-L-Phe-*Z*-ΔPhe-OMe•TFA, **5**, (2.4 equiv., 0.60 mmol, 0.26 g) were sequentially added under a nitrogen atmosphere. This mixture was stirred for 1 h at room temperature and then heated under reflux for 48 h at 80 °C. The white precipitate was filtered and washed several times with cold water (25 mL, to remove the by-product Et_3_NHCl) and finally with diethyl ether (25 mL). This white solid was dried under vacuum to provide compound **9** as a white solid (84 mg, 0.11 mmol, 44%). ^1^H-NMR (400 MHz, DMSO-d_6_, δ): 3.03–3.09 (2H, dd, *J* = 3.9 and 13.8 Hz, β-C*H*_A_H_B_); 3.21–3.24 (2H, dd, *J* = 10.8 and 12.8 Hz, β-CH_A_*H*_B_); 3.70 (6H, s, OC*H*_3_); 4.83–4.89 (2H, m, α-C*H*); 7.16–7.35 (18H, m, Ar*H* and β-C*H* ∆Phe); 7.68 (4H, m, Ar*H*); 7.88 (4H, s, Ar*H* central ring); 8.84 (2H, d, *J* = 8.0 Hz, α-N*H* Phe); 9.96 (2H, s, α-N*H* ∆Phe) ppm. ^13^C-NMR (100.6 MHz, DMSO-d^6^, δ): 36.4 (CH_2_, β-*C*H_2_ Phe), 52.2 (CH_3_, O*C*H_3_), 55.2 (CH, α-*C*H Phe), 125.9 (C α-*C* ∆Phe), 126.4 (CH), 127.4 (CH), 128.1 (CH), 128.6 (CH), 129.2 (CH), 129.5 (CH), 130.1 (CH), 132.0 (CH, β-C*H* ∆Phe), 133.3 (C), 136.3 (C), 138.3 (C), 165.4 (C, *C*=O), 165.9 (C, *C*=O), 171.5 (C, *C*=O) ppm. HRMS (ESI): *m*/*z*: [M + H]^+^ calcd. for C_46_H_42_N_4_O_8_ 779.30026; found 779.30759.

#### 4.1.2. Compound **1**

Compound **9** (0.11 mmol, 84.0 mg) was dissolved in 1,4-dioxane (3.21 mL) and NaOH (1 M) (3.0 equiv., 0.32 mL). The reaction was monitored by TLC. When the starting material was consumed (typically 4 h), the organic solvent was removed under reduced pressure, and the reaction mixture was acidified to pH 3 with KHSO_4_ (1 M). The solid precipitate was filtered, affording compound **1** (66.0 mg, 0.09 mmol, 82%), as a white solid. ^1^H-NMR (400 MHz, DMSO-d_6_, δ): 3.02–3.08 (2H, dd, *J* = 10.8 and 13.7 Hz, β-C*H*_A_H_B_); 3.21–3.24 (2H, dd, *J* = 3.6 and 13.7, β-CH_A_*H*_B_); 4.84–4.89 (2H, m, α-C*H*); 7.14–7.33 (14H, m, Ar*H* and β-C*H* ∆Phe); 7.40–7.42 (4H, d, *J* = 7.2 Hz, Ar*H* Phe); 7.65–7.67 (4H, m, Ar*H* ∆Phe); 7.85–7.87 (4H, m, Ar*H* central ring); 8.79–8.81 (2H, d, *J* = 8.4 Hz; α-N*H* Phe); 9.79 (2H, s, α-N*H* ∆Phe), 12.7 (2H, s, CO_2_*H*) ppm. ^13^C-NMR (100.6 MHz, DMSO-d_6_: δ): 36.5 (CH_2_, β-*C*H_2_ Phe), 55.2 (CH, α-*C*H Phe), 126.6 (C, α-*C* ∆Phe), 126.3 (CH), 127.3 (CH), 128.1 (CH), 128.5 (CH), 129.2 (CH), 129.2 (CH), 130.0 (CH), 131.9 (CH, β-*C*H ∆Phe), 133.6 (C), 136.3 (C), 138.4 (C), 165.8 (C, *C*=O), 166.2 (C, *C*=O), 171.1 (C, *C*=O) ppm. HRMS (ESI): *m/z*: [M + H]^+^ calcd. for C_44_H_38_N_4_O_8_ 751.26896; found 751.27670.

#### 4.1.3. Compound **10**

To a stirred suspension of terephthaloyl chloride (**7**) (1.0 equiv., 0.244 mmol, 0.050 g) in dry THF (5 mL), Et_3_N (5.0 equiv., 1.22 mmol, 0.17 mL) and H-L-Tyr-*Z*-ΔPhe-OMe•TFA, **6**, (2.4 equiv., 0.586 mmol, 0.266 g) were sequentially added under a nitrogen atmosphere. This mixture was stirred for 1 h at room temperature and then heated under reflux for 48 h at 80 °C. The white precipitate was filtered and washed several times with cold water (25 mL, to remove the by-product Et_3_NHCl) and finally with diethyl ether (25 mL). This white solid was dried under vacuum to provide compound **18** as a yellow solid (145 mg, 0.178 mmol, 73%). ^1^H-NMR (400 MHz, DMSO-d_6_, δ): 2.87–2.97 (2H, app.t, β-C*H*_A_H_B_ Tyr), 3.03–3.13 (2H, app.t, β-CH_A_*H*_B_ Try), 3.70 (6H, s, OC*H*_3_), 4.75–4.80 (2H, m, α-C*H* Tyr), 6.65 (4H, d, *J* = 8.0 Hz, Ar*H* Tyr), 7.19 (4H, d, *J* = 8.0 Hz, Ar*H* Tyr), 7.26 (2H, s, β-C*H* ΔPhe), 7.34–7.35 (6H, m, Ar*H* ΔPhe), 7.66 (4H, s br., Ar*H* ΔPhe), 7.90 (4H, s, Ar*H* central ring), 8.77 (2H, d, *J* = 8 Hz, N*H* Tyr), 9.18 (2H, s, O*H*), 9.92 (2H, s, N*H* ΔPhe) ppm. ^13^C-NMR (100.6 MHz, DMSO-d_6_, δ): 35.7 (CH_2_, β-*C*H_2_ Tyr), 52.2 (CH_3_, O*C*H_3_), 55.6 (CH, α-*C*H Tyr), 115.0 (CH, Tyr), 126.0 (C), 127.4 (CH, central ring), 128.3 (C, *C*-Tyr), 128.5 (CH, *C*H ΔPhe), 129.5 (CH, *C*H ΔPhe), 130.1 (CH, *C*H ΔPhe), 130.1 (CH, *C*H Tyr), 132.0 (CH, β-*C*H ∆Phe), 133.3 (C, *C*-ΔPhe), 136.3 (C, central ring), 155.8 (C, Tyr *C*-OH), 165.4 (C, *C*=O), 165.8 (C, *C*=O), 171.6 (C, *C*=O) ppm. HRMS (ESI): *m*/*z*: [M + H]^+^ calcd. for C_46_H_42_N_4_O_10_ 811.2901; found 811.2956.

#### 4.1.4. Compound **2**

Compound **10** (0.178 mmol, 145 mg) was dissolved in 1,4-dioxane (3.60 mL) and NaOH (1M) (3.0 equiv., 0.40 mL). The reaction was monitored by TLC. When the starting material was consumed (typically 4 h), the organic solvent was removed under reduced pressure, and the reaction mixture was acidified to pH 3 with KHSO_4_ (1 M). The solid precipitate was filtered to afford compound **2** (113 mg, 0.14 mmol, 81%), as a white solid. ^1^H-NMR (400 MHz, DMSO-d_6_, δ): 2.90–2.96 (2H, app.t, β-C*H*_A_H_B_ Tyr), 3.10–3.13 (2H, app.t, β-CH_A_*H*_B_ Tyr), 4.75–4.81 (2H, m, α-C*H* Tyr), 6.64 (4H, d, *J* = 8 Hz, Ar*H* Tyr), 7.19 (4H, d, *J* = 8 Hz, Ar*H* Tyr), 7.29 (2H, s, β-C*H* ΔPhe), 7.32–7.33 (6H, m, Ar*H* ΔPhe), 7.63–7.66 (4H, m, Ar*H* ΔPhe), 7.88 (4H, d *J* = 4.0 Hz, Ar*H* central ring), 8.73 (2H, d, *J* = 8.0 Hz, N*H* Tyr), 9.17 (2H, s, O*H*), 9.74 (2H, s, N*H* ΔPhe), 12.5 (2H, s, CO_2_*H*) ppm. ^13^C-NMR (100.6 MHz, DMSO-d_6_: δ): 35.8 (CH_2_, β-*C*H_2_ Tyr), 55.6 (CH, α-*C*H Tyr), 115.0 (CH, *C*H Tyr), 126.7 (C), 127.4 (CH, central ring), 128.4 (C, *C*-Tyr), 128.5 (CH, *C*H ΔPhe), 129.2 (CH, *C*H ΔPhe), 130.0 (CH, *C*H ΔPhe), 130.2 (CH, *C*H Tyr), 131.7 (CH, β-*C*H ∆Phe), 133.7 (C, *C*-ΔPhe), 136.4 (C, central ring), 155.8 (C, Tyr *C*-OH), 165.8 (C, *C*=O), 166.3 (C, *C*=O), 171.3 (C, *C*=O) ppm. HRMS (ESI): *m*/*z*: [M + H]^+^ calcd. for C_44_H_38_N_4_O_10_ 783.2588; found 783.2661.

#### 4.1.5. Compound **11**

Naphthalene-2,6-dicarboxylic acid (**8**) (1.0 equiv., 0.53 mmol, 0.11 g) was dissolved in MeCN (6 mL) and cooled to 0 °C. H-L-Phe-*Z*-ΔPhe-OMe•TFA (2.2 equiv., 1.16 mmol, 0.51 g), Et_3_N (6.0 equiv., 3.17 mmol, 0.44 mL), and HBTU (2.4 equiv., 1.67 mmol, 0.48 g) were added sequentially, with 2 min between each addition, and the mixture was stirred at rt overnight. The solvent was removed under reduced pressure to afford a residue that was partitioned between EtOAc (50 mL) and KHSO_4_ (1 M, 50 mL). After separation of the phases, the organic phase was thoroughly washed with KHSO_4_ (1 M, 3 × 50 mL), NaHCO_3_ (1 M, 3 × 50 mL), and brine (3 × 50 mL) and then dried with MgSO_4_. Filtration followed by removal of the solvent under reduced pressure afforded compound **11** as a white solid (0.420 g, 0.510 mmol, 96%). ^1^H-NMR (400 MHz, DMSO-d_6_, δ): 3.11–3.17 (2H, app. t, β-C*H*_A_H_B_ Phe), 3.24–3.29 (2H, dd, *J* = 3.6 and 14 Hz, β-CH_A_*H*_B_ Phe), 3.71 (6H, s, OC*H*_3_), 4.90–4.96 (2H, m, α-C*H* Phe), 7.16–7.47 (18H, m, Ar*H* Phe), 7.69–7.72 (4H, m, Ar*H* ΔPhe), 7.97 (2H, d, *J* = 8.8 Hz, Ar*H_3_* central ring), 8.10 (2H, d, *J* = 8.8 Hz, Ar*H_4_* central ring), 8.50 (2H, s, Ar*H_1_* central ring), 9.00 (2H, d, *J* = 6.8 Hz, α-N*H* Phe), 10.0 (2H, s, α-N*H* ΔPhe) ppm. ^13^C-NMR (100.6 MHz, DMSO-d_6_: δ): 36.5 (CH_2_, β-*C*H_2_ Phe), 52.2 (CH_3_, O*C*H_3_), 55.3 (CH, α-*C*H Phe), 125.1 (CH, *C_3_*H central ring), 125.9 (C), 126.3 (CH), 127.6 (CH, *C_1_*H central ring), 128.1 (CH), 128.5 (CH), 128.9 (CH, *C_4_*H central ring), 129.2 (CH), 129.5 (CH), 130.1 (CH, *C*H ΔPhe), 132.1 (CH), 132.7 (C), 133.2 (C), 138.3 (C), 165.3 (C, *C*=O), 166.3 (C, *C*=O), 174.6 (C, *C*=O) ppm. HRMS (ESI): *m*/*z*: [M + H]^+^ calcd. for C_50_H_44_N_4_O_8_ 829.3159; found 829.3228.

#### 4.1.6. Compound **3**

Compound **11** (0.51 mmol, 0.42 g) was dissolved in 1,4-dioxane (15 mL) and NaOH (1 M) (3.00 equiv., 1.52 mL). The reaction was monitored by TLC. When the starting material was consumed (typically 4 h), the organic solvent was removed under reduced pressure, and the reaction mixture was acidified to pH 3 with KHSO_4_ (1 M). The solid precipitate was filtered to afford compound **3** (0.400 g, 0.500 mmol, 98%), as a white solid. ^1^H-NMR (400 MHz, DMSO-d_6_, δ): 3.10–3.17 (2H, dd, *J* = 3.12 and 13.2 Hz, β-C*H*_A_H_B_ Phe), 3.26–3.29 (2H, app. t, β-CH_A_*H*_B_ Phe), 4.91–4.97 (2H, m, α-C*H* Phe), 7.14–7.45 (18H, m, Ar*H* Phe), 7.65–7.67 (4H, m, Ar*H* ΔPhe), 8.05 (2H, d, *J* = 8.4 Hz, Ar*H_3_* central ring), 7.95 (2H, d, *J* = 8.4 Hz, Ar*H_4_* central ring, 8.46 (2H, s, Ar*H_1_* central ring), 8.89 (2H, d, *J* = 6.8 Hz, N*H* Phe), 9.76 (2H, s, N*H* ΔPhe), 12.6 (2H, s, CO_2_*H*) ppm. ^13^C-NMR (100.6 MHz, DMSO-d_6_ δ): 36.6 (CH_2_, β-*C*H_2_ Phe), 55.4 (CH, α-*C*H Phe), 125.1 (CH, *C_3_*H central ring), 125.9 (C), 126.3 (C), 126.9 (CH), 127.5 (CH, *C_1_*H central ring), 128.1 (CH), 128.4 (CH), 128.9 (CH, *C_4_*H central ring), 129.0 (CH), 129.1 (CH), 129.2 (CH), 130.0 (CH), 131.4 (CH), 132.8 (C), 133.2 (C), 133.8 (C), 138.4 (C), 166.3 (C, 2 × *C*=O), 171.1 (*C*=O) ppm. HRMS (ESI): *m*/*z:* [M + H]^+^ calcd. for C_48_H_44_N_4_O_8_ 801.2846; found 801.2925.

#### 4.1.7. Compound **12**

Naphthalene-2,6-dicarboxylic acid (**8**) (1.0 equiv., 0.23 mmol, 0.05 g) was dissolved in DMF (5 mL) and cooled to 0 °C. H-L-Tyr-*Z*-ΔPhe-OMe•TFA (2.4 equiv., 0.56 mmol, 0.25 g), Et_3_N (6.0 equiv., 1.40 mmol, 0.10 mL), and HBTU (2.4 equiv., 0.56 mmol, 0.21 g) were added sequentially, with 2 min between each addition, and the mixture was stirred at rt for 2 days. The solvent was removed under reduced pressure to afford a residue that was partitioned between EtOAc (50 mL) and KHSO_4_ (1M, 50 mL). After separation of the phases, the organic phase was thoroughly washed with KHSO_4_ (1 M, 2 × 50 mL) and brine (3 × 50 mL) and then dried with MgSO_4_. Filtration followed by removal of the solvent under reduced pressure afforded compound **12** as a white solid (196 mg, 0.228 mmol, 98%). ^1^H-NMR (400 MHz, DMSO-d_6_, δ): 3.02–3.08 (2H, dd, *J* = 10.8 and 13.6 Hz, β-C*H*_A_H_B_ Tyr), 3.12–3.16 (2H, dd, *J* = 4.0 and 14.0 Hz, β-CH_A_H_B_ Tyr), 3.70 (6H, s, OC*H*_3_), 4.79–4.85 (2H, m, α-C*H* Tyr), 6.68 (4H, d, *J* = 8.4 Hz, Ar*H* Tyr), 7.23 (4H, d, *J* = 8.4 Hz, Ar*H* Tyr), 7.26 (2H, s, β-C*H* ΔPhe), 7.27–7.30 (6H, m, Ar*H* ΔPhe), 7.68–7.70 (4H, m, Ar*H* ΔPhe), 8.00 (2H, d, *J* = 9.6 Hz, Ar*H_3_* central ring), 8.07 (2H, d, *J* = 8.8 Hz, Ar*H_4_* central ring), 8.54 (2H, s, Ar*H_1_* central ring), 9.01 (2H, d, *J* = 8.4 Hz, N*H* Tyr), 9.29 (2H, s, O*H*), 10.1 (2H, s, N*H* ΔPhe) ppm. ^13^C-NMR (100.6 MHz, DMSO-d_6_ δ): 35.8 (CH_2_, β-*C*H_2_ Tyr), 52.2 (CH_3_, O*C*H_3_), 55.9 (CH, α-*C*H Tyr), 115.0 (CH, *C*H Tyr), 125.1 (CH, *C_3_*H central ring), 126.0 (C), 127.6 (CH, *C_1_*H central ring), 128.2 (C), 128.5 (CH, *C*H ∆Phe), 128.8 (CH, *C-4*, central ring), 129.4 (CH, *C*H ∆Phe), 130.1 (CH, *C*H ΔPhe), 130.2 (CH, *C*H Try), 132.1 (CH, β-*C*H ∆Phe), 132.7 (C), 133.2 (C), 133.3 (C), 155.9 (C), 165.4 (C, *C*=O), 166.2 (C, *C*=O), 171.8 (C, *C*=O) ppm. HRMS (ESI): *m*/*z*: [M + Na]^+^ calcd. for C_50_H_44_N_4_O_10_ 883.3057; found 883.2961.

#### 4.1.8. Compound **4**

Compound **12** (0.228 mmol, 196 mg) was dissolved in 1,4-dioxane (7 mL) and NaOH (1 M) (3.00 equiv., 0.7 mL). The reaction was monitored by TLC. When all the starting material was consumed (typically 4 h), the organic solvent was removed under reduced pressure, and the reaction mixture was acidified to pH 3 with KHSO_4_ (1 M). The solid precipitate was filtered to afford compound **4** (188 mg, 0.225 mmol, 98%), as a white solid. ^1^H-NMR (400 MHz, DMSO-d_6_, δ): 3.10–3.17 (2H, app.t, β-C*H*_A_H_B_ Tyr), 3.26–3.29 (2H, dd, *J* = 3.6 and 14.0 Hz, β-CH_A_*H*_B_ Tyr), 4.81–4.87 (2H, m, α-C*H* Tyr), 6.66 (4H, d, *J* = 8.8 Hz, Ar*H* Tyr), 7.22 (4H, d, *J* = 8.4 Hz, Ar*H* Tyr), 7.29 (2H, s, β-C*H* ΔPhe), 7.31–7.34 (6H, m, Ar*H* ΔPhe), 7.66–7.67 (4H, m, Ar*H* ΔPhe), 7.96 (2H, d, *J* = 9.2 Hz, Ar*H_3_* central ring), 8.06 (2H, d, *J* = 8.4 Hz, Ar*H_4_* central ring), 8.47 (2H, s, Ar*H_1_* central ring), 8.84 (2H, d, *J* = 10 Hz, N*H* Tyr), 9.15 (2H, s, O*H*), 9.77 (2H, s, N*H* ΔPhe), 12.3 (2H, s, CO_2_*H*) ppm. ^13^C-NMR (100.6 MHz, DMSO-d_6_ δ): 35.8 (CH_2_, β-*C*H_2_ Phe), 55.8 (CH, α-*C*H Phe), 114.9 (CH, *C*H Tyr), 125.1 (CH, *C_3_*H central ring), 126.7 (C), 127.6 (CH, *C_1_*H central ring), 128.3 (CH), 128.4 (CH, CH ΔPhe), 128.8 (CH, *C_4_*H central ring), 129.2 (CH, *C*H ΔPhe), 130.0 (CH, *C*H ΔPhe), 130.1 (CH, *C*H Try), 131.9 (CH, β-*C*H ∆Phe), 132.8 (C), 133.2 (C), 133.6 (C), 155.8 (C), 166.2 (C, 2× *C*=O), 171.4 (C, *C*=O) ppm. HRMS (ESI): *m*/*z*: [M + H]^+^ calcd. for C_48_H_40_N_4_O_10_ 833.2744; found 833.2819.

### 4.2. Hydrogel Preparation

Dehydrodipeptides **1**, **2** and **3** were weighed into sample vials, water was added, and the suspension was adjusted under magnetic stirring to *circa* pH 10 (pH meter) by the addition of NaOH (1 M), and D-glucono-δ-lactone (GDL) was added. The solutions were left standing overnight at room temperature (20–25 °C).

### 4.3. Rheology

The viscoelastic characterization of hydrogels was performed with a stress-controlled rotational rheometer Anton Paar MCR300 (Anton Paar GmbH, Graz, Austria). Gel-forming solutions were loaded in the shearing geometry (a Couette cell with 1 mL volume and 0.5 mm gap) at 25 °C. The liquid sample was pre-sheared at a shear rate of 5 s^−1^ during one minute to homogenize the sample in the shearing geometry. Then, the gelation kinetics was monitored during 10 h by applying a small amplitude (0.001%) oscillatory shear at 1 Hz and recording both storage (G′) and loss (G″) moduli at each second. Then, mechanical spectra were recorded, and gels were submitted to a heating–cooling cycle between 25 and 40 °C at a rate of +/−1 °C/min. Finally, gels were submitted to a strain sweep.

### 4.4. Molecular Dynamics

The molecular structure of the compounds was designed with the program GaussView, and optimized geometries of the ground state were obtained from ab initio molecular quantum chemistry calculations, with Gaussian 09 software [36]. Parameterization was carried out using parameters from the natural amino acids in the GROMOS 54a7 force field [37,38]. To validate the proposed parameters, the residues of dehydrophenylalanine and 2,6-naphthalenedicarboxylic acid were subjected to 12,000 steps of energy minimization calculations with the steepest descent algorithm and 100 ps MD simulation in a cubic box solvated with Simple Point Charge (SPC) water model [39]. Validation was carried out by analyzing the convergence of the system’s potential energy and the geometry of the new residues. All simulations were run with the GROMACS 5.1.4 software package [40]. The compounds were posteriorly modeled using MARTINI coarse-grained (CG) model [41,42], and negative charges were assigned to main-chain beads at C terminal, as it is the most prevalent charge state at pH 7, and the system was neutralized by adding sodium ions. Then, the dehydropeptides were randomly placed in an 8 × 8 × 8 nm cubic box with a minimum distance of 3 Å and solvated in standard MARTINI CG water (four water molecules per bead) for different final concentrations (≈0.08 M, ≈0.16 M, and ≈0.32 M). The high concentration enables accelerating the assembly process. The CG simulations were carried out with MARTINI force field (version 2.2) [41]. The temperature (τT = 1 ps) and pressure (τT = 3 ps) were kept constant at 303 K at 1 bar, respectively, using the Berendsen algorithms [43]. The bonds lengths of peptides side chains and 2,6-naphthalenedicarboxylic ring were constrained with the LINCS algorithm [44]. For the treatment of long-range interactions, the reaction-field method was employed in the range 0.0–1.1 nm, and a relative dielectric constant εr=15 was used in standard CG water simulations for screening of electrostatic interactions, and the non-bonded Lennard-Jones interactions were modified using Gromacs’ potential-shift-Verlet option with a cut-off of 1.1 nm. The systems were energy minimized with the steepest descent integrator for 5000 steps using 25 fs time steps, and an NPT ensemble run over 40 × 106 steps (1 µs) was performed [24,27], which equates to 4 µs “effective time” due to the smoothness of the CG potentials [41,45]. All times presented in the results are expressed as the mentioned effective time. The aggregation properties of each peptide system were evaluated by identifying the occurrence of peptide clusters formed in the simulation box using a cut-off of 0.5 nm between the center of mass of each peptide [46]. Visualization of the aggregates was analyzed with the PyMOL software. The simulated systems were also analyzed based on their aggregation propensity (AP score), which is defined as the ratio between the solvent-accessible surface area (SASA) of the initial randomized state and the final configuration of the simulation [27].

### 4.5. CD Spectroscopy

CD spectra were recorded under a constant flor of N_2_ using a spectropolarimeter Jasco model J-1500 (JASCO, Tokyo, Japan) at 25 °C using solutions of hydrogelators **1**, **2** and **3** (0.01 wt %). The solutions of the hydrogelators were loaded into 0.1 mm quartz cells.

### 4.6. Scanning Transmission Electron Microscopy (STEM)

STEM images were recorded using a NanoSEM—FEI Nova 200 (FEI Technologies, Inc., Hillsboro, OR, USA), operating at 15 kV, coupled to an Electron-Dispersive Spectroscopic analyzer (EDS) and Electron Backscatter Diffraction EDAX—Pegasus X4M analyzer and detection system (EBSD) at SEMAT (Serviços de Caracterização de Materiais), Guimarães, Portugal. After preparation of the hydrogel, a small portion of each sample was placed onto a TEM 400 mesh copper grid with Formvar/Carbon, held by tweezers, and the excess solution was cleaned. The processing of STEM images was performed using ImageJ software (National Institutes of Health (NIH), Bethesda, MD, USA), which consisted in enhancing local contrast and adjusting brightness followed by the manual selection of fibers.

### 4.7. Sustained Release Assays

Hydrogels of **1**, **2**, and **3** were prepared as described in Section 4.2 to form 1 mL hydrogels containing the same concentration of the hydrogelators described above and the appropriate cargo (methylene blue (0.1 nM), methyl orange (0.2 nM), or ciprofloxacin (0.2 nM)), in a slightly modified version of the procedure described by Abraham et al. [1]. After allowing to stand overnight, 1.5 mL of water was carefully added to the surface of the hydrogels. Aliquots of the layered solution (100 μL) were removed at 1 h, 2 h, 3 h, 4 h, 6 h, 24 h, 72 h, and 6 days from the time the water was initially layered on top of the hydrogel. After removing each aliquot, the volume water was immediately replaced by an equal volume of water. The concentration of methylene blue or methyl orange in each aliquot was determined by measuring the absorbance at λ_max_ of the dye (666 nm for methylene blue and 465 nm for methyl orange) using a microplate reader and then converting the value to percentage release (using a standard calibration curve). The concentration of ciprofloxacin in each aliquot was determined using analytical HPLC, where the integrated peak area was converted to a percentage release (using a standard calibration curve). Each experiment was performed in triplicate, and the mean percentage cargo release was plotted against time.

### 4.8. Cell Culture

Human keratinocytes cell line HaCaT was from ATCC. Cells were cultured in DMEM supplemented with 10% FBS and 1% penicillin/streptomycin and were incubated at 37 °C, in a humidified atmosphere of 5% CO_2_.

### 4.9. MTT Assay/LDH Leakage

Cells were seeded in 96-well plates (1.5 × 10^4^ cells/well) and left to attach for 24 h. After this period, cells were incubated with different concentrations of the molecules under study for another 24 h. Then, cell viability was evaluated based on the ability of metabolically active cells to convert MTT to formazan over the course of 2 h. Absorbances were measured at 570 nm in a Multiskan GO plate reader (Thermo Fisher Scientific; Waltham, MA, USA) and results were expressed as percentage of the respective control and correspond to the mean ± standard error of the mean (SEM) of at least three independent experiments performed in triplicate. On the other hand, to assess the release of the stable cytosolic enzyme lactate dehydrogenase (LDH) into the media, 24 h after the incubation of the cells with the different concentrations of the molecules under study, 40 µL of culture media were removed to a 96-well plate. LDH released was determined using a CytoTox 96^®^ assay kit (Promega; Madison, WI, USA) according to the manufacturer’s instructions. Then, 1% Triton X-100 was used as positive control to assure cell lysis (30 min). Absorbances were measured at 490 nm in a Multiskan GO plate reader (Thermo Fisher Scientific; Waltham, MA, USA), and results correspond to the fold-increase of absorbance in treated vs. untreated cells of three independent experiments performed in duplicate. Following assessment of the distribution of the results, ANOVA was performed using GraphPad Prism 8.0 (GraphPad Prism Inc., San Diego, CA, USA).

### 4.10. DNA/Protein Quantification

Cells were cultured at the same density described above for the MTT assay, in the presence of the molecules under study. After incubation, the culture medium was replaced by 50 μL of ultra-pure water, plates being incubated for 30 min at 37 °C and immediately frozen at −80 °C. DNA/protein quantification was performed in a triplicate pool using a Qubit^TM^ dsDNA HS/Protein Assay Kit according to manufacturer’s instructions. Results are expressed as percentage of the respective control and correspond to the mean ± SEM of two independent experiments performed in triplicate. Following assessment of the distribution of the results, ANOVA was performed (GraphPad Prism 8.0).

## Figures and Tables

**Table 1:** Gelation conditions for peptides **1**–**4**.

Peptide	Critical Gelation Concentration (cgc)		GDL Concentration (wt %)	pH	cLogP *
	wt %	mM			
**1**	0.3	4.0	0.4	5.10	5.82
**2**	0.4	5.1	0.4	4.15	4.49
**3**	0.3	3.7	0.4	5.30	6.69
**4**	No hydrogel				5.55

* cLogP value obtained from https//molinspiration.com, accessed 20 March 2021.

**Table 2:** Parameters from the Saitô’s equation computed from separate fits to the kinetics.

Compound	*G*0 (Pa)	*dG* (Pa)	*k_n_* (h^−1^)	*k* (h^−1^)	*R* ^2^
**1**	0 ± 57	2392 ± 66	0.40 ± 0.04	0.18 ± 0.10	0.89
**2**	0 ± 25	5722 ± 39	0.02 ± 0.001	1.84 ± 0.04	0.99

**Table 3:** G′ and G″ for hydrogel **1**, **2**, and **3**.

Hydrogel	G′ (Pa)	G″ (Pa)
**1**	2.5 × 10^3^	6.5 × 10^1^
**2**	4.5 × 10^3^	1.4 × 10^2^
**3**	3.7 × 10^2^	1.3 × 10^1^

**Table 4:** Percentage release of cargo from hydrogels **1**–**3** after 6 days.

Hydrogelator	MB Released (%)	MO Released (%)	Ciprofloxacin Released (%)
**1**	12.1	60.9	32.3
**2**	7.63	96.2	58.8
**3**	9.55	60.3	20.3

**Table 5:** Release coefficients of the Korsmeyer–Peppas model obtained for methyl orange and ciprofloxacin release profiles in hydrogels **1**, **2**, and **3**.

Cargo	Hydrogel	*K*	*n*	*R* ^2^
**Methyl Orange**	**1**	0.0200	0.4000	0.9509
	**2**	0.0900	0.2700	0.9785
	**3**	0.0400	0.3300	0.9497
**Ciprofloxacin**	**1**	0.0200	0.3300	0.8677
	**2**	0.0030	0.5800	0.8956
	**3**	0.0001	0.6300	0.9406

## Data Availability

Not applicable.

## Supplementary Materials

The following are available online at https://www.mdpi.com/article/10.3390/gels7020052/s1, Figure S1: Optical images of hydrogels formed by hydrogelators **1** (A), **2** (B), **3** (C). Figure S2: Snapshot at different time points of the self-assembly of compound 3 over a 4 µs long CG MD simulation in different concentrations Figure S3: Snapshot at different time points of the self-assembly of compound **4** over a 4 µs long CG MD simulation in different concentrations. Figure S4: Scanning transmission electron microscopy (STEM) images of Cbz-Phe-ΔPhe-OH and Cbz-TyrΔPhe-OH at 0.3 wt %, at different magnifications (4, 2, and 1 µm). Figure S5: Elastic and viscous modulus during the kinetic process of gelation of compounds **1**, **2**, and **3**. Figure S6. Frequency dependence of the shear elastic G′ (empty symbols) and loss G″ (filled symbols) moduli for the compounds **1**, **2**, and **3**. Figure S7: Thermal variation of the storage G′ recorded at a frequency of 1 Hz with a strain amplitude of 0.005%, during a heating and cooling cycle performed at a rate of 1 °C/min. Figure S8: DNA and protein content of HaCaT cells in the same conditions as Figure 8. Figure S9: LDH activity found in the culture media of HaCaT cells treated with 1–4 for 24 h at the concentrations presented. Triton X-100 was used as positive control to lyse cells. Figure S10: Calibration curve to determine the amount of cargo in present in the layered solution above the hydrogel. Figure S11: Percentage of cargo release vs. time over 6 days. Release of methylene blue, methyl orange, and ciprofloxacin from hydrogels of **1**, **2**, and **3**. Figure S12: Data to Korsmeyer–Peppas Model to describe the release kinetics of methyl orange from hydrogels **1**, **2**, and **3**. Figure S13: Data to Korsmeyer–Peppas Model to describe the release kinetics of ciprofloxacin from hydrogels **1**, **2**, and **3**. Table S1: Aggregation propensity score (AP) values obtained for the various simulations of compound **3** and **4**, and final SASA fraction of the dehydrophenylalanine (ΔPhe), tyrosine (Tyr), phenylalanine (Phe), and 2,6-naphthalenedicarboxyl (Naph) groups.

## References

[1] Abraham B.L., Toriki E.S., Tucker N.D.J., Nilsson B.L. (2020). Electrostatic interactions regulate the release of small molecules from supramolecular hydrogels. J. Mater. Chem. B.

[2] Song Y., Gao J., Xu X., Zhao H., Xue R., Zhou J., Hong W., Qiu H. (2017). Fabrication of thermal sensitive folic acid based supramolecular hybrid gels for injectable drug release gels. Mater. Sci. Eng. C.

[3] Jayawarna V., Richardson S.M., Hirst A.R., Hodson N.W., Saiani A., Gough J.E., Ulijn R.V. (2009). Introducing chemical functionality in Fmoc-peptide gels for cell culture. Acta Biomater..

[4] Zhao H., Liu M., Zhang Y., Yin J., Pei R. (2020). Nanocomposite hydrogels for tissue engineering applications. Nanoscale.

[5] Yadav N., Chauhan M.K., Chauhan V.S. (2020). Short to ultrashort peptide-based hydrogels as a platform for biomedical applications. Biomater. Sci..

[6] Adams D.J. (2011). Dipeptide and Tripeptide Conjugates as Low-Molecular-Weight Hydrogelators. Macromol. Biosci..

[7] Jervis P.J., Amorim C., Pereira T., Martins J.A., Ferreira P.M.T. (2020). Exploring the properties and potential biomedical applications of NSAID-capped peptide hydrogels. Soft Matter.

[8] Arokianathan J.F., Ramya K.A., Janeena A., Deshpande A.P., Ayyadurai N., Leemarose A., Shanmugam G. (2020). Non-proteinogenic amino acid based supramolecular hydrogel material for enhanced cell proliferation. Colloids Surf. B Biointerfaces.

[9] Piepenbrock M.-O.M., Lloyd G.O., Clarke N., Steed J.W. (2010). Metal- and Anion-Binding Supramolecular Gels. Chem. Rev..

[10] Draper E.R., Adams D.J. (2019). Controlling the Assembly and Properties of Low-Molecular-Weight Hydrogelators. Langmuir.

[11] Yang Z., Liang G., Ma M., Gao Y., Xu B. (2007). Conjugates of naphthalene and dipeptides produce molecular hydrogelators with high efficiency of hydrogelation and superhelical nanofibers. J. Mater. Chem..

[12] Vilaça H., Hortelão A.C.L., Castanheira E.M.S., Queiroz M.-J.R.P., Hilliou L., Hamley I.W., Martins J.A., Ferreira P.M.T. (2015). Dehydrodipeptide Hydrogelators Containing Naproxen N-Capped Tryptophan: Self-Assembly, Hydrogel Characterization, and Evaluation as Potential Drug Nanocarriers. Biomacromolecules.

[13] Vilaça H., Pereira G., Castro T.G., Hermenegildo B.F., Shi J., Faria T.Q., Micaêlo N., Brito R.M.M., Xu B., Castanheira E.M.S. (2015). New self-assembled supramolecular hydrogels based on dehydropeptides. J. Mater. Chem. B.

[14] Carvalho A., Gallo J., Pereira D.M., Valentão P., Andrade P.B., Hilliou L., Ferreira P.M.T., Bañobre-López M., Martins J.A. (2019). Magnetic Dehydrodipeptide-Based Self-Assembled Hydrogels for Theragnostic Applications. Nanomaterials.

[15] Veloso S.R.S., Magalhães C.A.B., Rodrigues A.R.O., Vilaça H., Queiroz M.-J.R.P., Martins J.A., Coutinho P.J.G., Ferreira P.M.T., Castanheira E.M.S. (2019). Novel dehydropeptide-based magnetogels containing manganese ferrite nanoparticles as antitumor drug nanocarriers. Phys. Chem. Chem. Phys..

[16] Vilaça H., Castro T., Costa F.M.G., Melle-Franco M., Hilliou L., Hamley I.W., Castanheira E.M.S., Martins J.A., Ferreira P.M.T. (2017). Self-assembled RGD dehydropeptide hydrogels for drug delivery applications. J. Mater. Chem. B.

[17] Veloso S.R.S., Jervis P.J., Silva J.F.G., Hilliou L., Moura C., Pereira D.M., Coutinho P.J.G., Martins J.A., Castanheira E.M.S., Ferreira P.M.T. (2021). Supramolecular ultra-short carboxybenzyl-protected dehydropeptide-based hydrogels for drug delivery. Mater. Sci. Eng. C.

[18] Hughes J.R., Miller A.S., Wallace C.E., Vemuri G.N., Lovine P.M. (2021). Biomedically Relevant Applications of Bolaamphiphiles and Bolaamphiphile-Containing Materials. Med. Pharm. Chem..

[19] Qiu F., Chen Y., Tang C., Zhou Q., Wang C., Shi Y.K., Zhao X. (2008). De Novo Design of a Bolaamphiphilic Peptide with only natural amino acids. Macromol. Biosci..

[20] Estroff L.A., Hamilton A.D. (2004). Water Gelation by Small Organic Molecules. Chem. Rev..

[21] Dou X., Li P., Zhang D., Feng C.L. (2012). C2-symmetric benzene-based hydrogels with unique layered structures for controllable organic dye adsorption. Soft Matter.

[22] Du X., Zhou J., Shi J., Xu B. (2015). Supramolecular Hydrogelators and Hydrogels: From Soft Matter to Molecular Biomaterials. Chem. Rev..

[23] Chakraborty P., Tang Y., Yamamoto T., Yao Y., Guterman T., Zilberzwige-Tal S., Adadi N., Ji W., Dvir T., Ramamoorthy A. (2020). Unusual two-step assembly of a minimalistic dipeptide-based functional hypergelator. Adv. Mater..

[24] Frederix P.W.J.M., Scott G.G., Abul-Haija Y.M., Kalafatovic D., Pappas C.G., Javid N., Hunt N.T., Ulijn R.V., Tuttle T. (2015). Exploring the sequence space for (tri-)peptide self-assembly to design and discover new hydrogels. Nat. Chem..

[25] Moreira I.P., Scott G.G., Ulijn R.V., Tuttle T. (2019). Computational prediction of tripeptide-dipeptide co-assembly. Mol. Phys..

[26] Guo C., Luo Y., Zhou R., Wei G. (2014). Triphenylalanine peptides self-assemble into nanospheres and nanorods that are different from the nanovesicles and nanotubes formed by diphenylalanine peptides. Nanoscale.

[27] Frederix P.W.J.M., Ulijn R.V., Hunt N.T., Tuttle T. (2011). Virtual screening for dipeptide aggregation: Toward predictive tools for peptide self-assembly. J. Phys. Chem. Lett..

[28] Liu J., Zheng H., Poh P.S.P., Machens H.-G., Schilling A.F. (2015). Hydrogels for Engineering of Perfusable Vascular Networks. Int. J. Mol. Sci..

[29] Adams D.J., Mullen L.M., Berta M., Chen L., Frith W.J. (2010). Relationship between molecular structure, gelation behaviour and gel properties of Fmoc-dipeptides. Soft Matter.

[30] Kamihira M., Naito A., Tuzi S., Saitô H., Nosaka A.Y. (2000). Conformational transitions and fibrillation mechanism of human calcitonin as studied by high-resolution solid-state 13C NMR. Protein Sci..

[31] Kini S., Bahadur D., Panda D. (2015). Mechanism of Anti-Cancer Activity of Benomyl Loaded Nanoparticles in Multidrug Resistant Cancer Cells. J. Biomed. Nanotechnol..

[32] Gupta M., Bagaria A., Mishra A., Mathur P., Basu A., Ramakumar S., Chauhan V.S. (2007). Self-Assembly of a Dipeptide- Containing Conformationally Restricted Dehydrophenylalanine Residue to Form Ordered Nanotubes. Adv. Mater..

[33] Mishra A., Panda J.J., Basu A., Chauhan V.S. (2008). Nanovesicles Based on Self-Assembly of Conformationally Constrained Aromatic Residue Containing Amphiphilic Dipeptides. Langmuir.

[34] Parween S., Misra A., Ramakumar S., Chauhan V.S. (2014). Self-assembled dipeptide nanotubes constituted by flexible β-phenylalanine and conformationally constrained α,β-dehydrophenylalanine residues as drug delivery system. J. Mater. Chem. B.

[35] Veloso S.R.S., Martins J.A., Hilliou L., Amorim C.O., Amaral V.S., Almeida B.G., Jervis P.J., Moreira R., Pereira D.M., Coutinho P.J.G. (2020). Dehydropeptide-based plasmonic magnetogels: A supramolecular composite nanosystem for multimodal cancer therapy. J. Mater. Chem. B.

[36] Frisch G.W., Trucks H.B., Schlegel G.E., Scuseria M.A., Robb J.R., Cheeseman G., Scalmani V., Barone B., Mennucci G.A., Petersson H. (2009). Gaussian 09, Revision, A.02.

[37] Huang W.L., Lin Z.X., van Gunsteren W.F. (2011). Rapid sampling of folding equilibria of β-Peptides in methanol using a supramolecular solvent model. J. Chem. Theory Comput..

[38] Schmid N., Eichenberger A.P., Choutko A., Riniker S., Winger M., Mark A.E., van Gunsteren W.F. (2011). Definition and testing of the GROMOS force-field versions 54A7 and 54B7. Eur. Biophys. J. Biophys. Lett..

[39] Berendsen H.J.C., Grigera J.R., Straatsma T.P. (1987). The missing term in effective pair potentials. J. Phys. Chem..

[40] Abraham M.J., van der Spoel D., Lindahl E., Hess B. (2016). The GROMACS Development Team. GROMACS User Manual Version 5.1.4. www.gromacs.org.

[41] Marrink S.J., Risselada H.J., Yefimov S., Tieleman D.P., de Vries A.H. (2007). The MARTINI force field: Coarse grained model for biomolecular simulations. J. Phys. Chem. B.

[42] Monticelli L., Kandasamy S.K., Periole X., Larson R.G., Tieleman D.P., Marrink S.J. (2008). The MARTINI coarse-grained force field: Extension to proteins. J. Chem. Theory Comput..

[43] Berendsen H.J.C., Postma J.P.M., van Gunsteren W.F., DiNola A., Haak J.R.J. (1984). Molecular dynamics with coupling to an external bath. Chem. Phys..

[44] Hess B., Bekker H., Berendsen H.J.C., Fraaije J. (1997). LINCS: A linear constraint solver for molecular simulations. J. Comput. Chem..

[45] Marrink S.J., de Vries A.H., Mark A.E. (2004). Coarse Grained Model for Semiquantitative Lipid Simulations. J. Phys. Chem. B.

[46] Szała B., Molski A. (2019). Aggregation kinetics of short peptides: All-atom and coarse-grained molecular dynamics study. Biophys. Chem..

