# Supplementary material for: Bolaamphiphilic Bis-Dehydropeptide Hydrogels as Potential Drug Release Systems
Source: Gels. 2021 Apr 29;7(2):52. doi: 10.3390/gels7020052 (PMC8162347; doi:10.3390/gels7020052)
Supplement: Supplementary file 1 [file gels-07-00052-s001.zip › gels-1176286-supplementary.pdf]

Supplementary Information

Bolaamphiphilic bis-dehydropeptide hydrogels as potential drug release systems

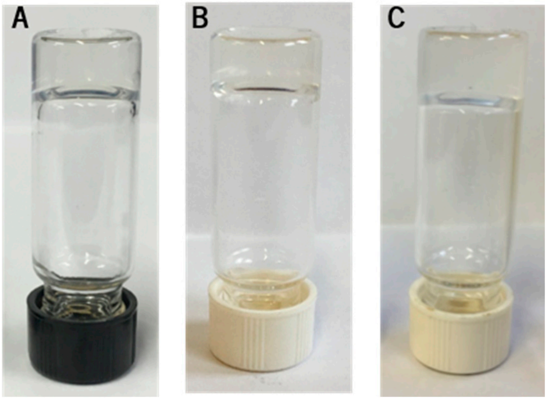

Figure S1: Optical images of hydrogels formed by hydrogelators **1** (A), **2** (B), **3** (C).

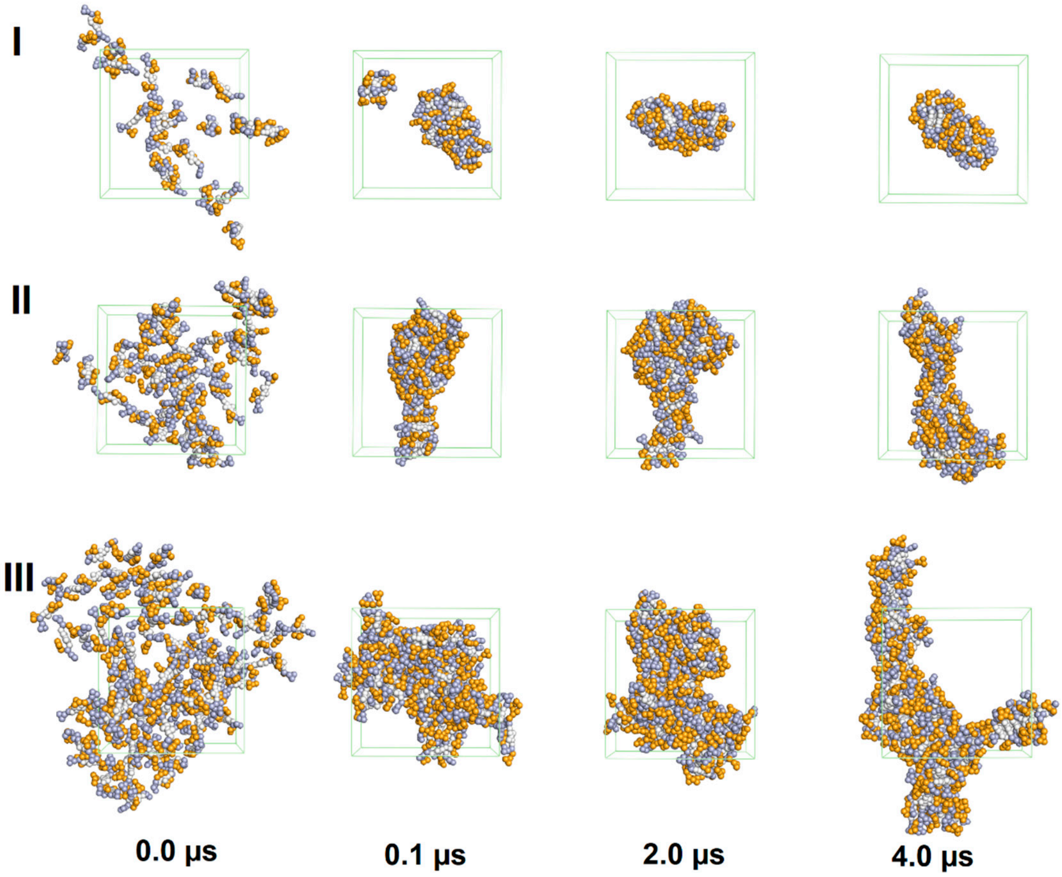

Figure S2. Snapshot at different time points of the self-assembly of compound **3** over a 4 μs long CG MD simulation in different concentrations: (I) 0.08 M; (II) 0.16 M; (III) 0.32 M. Water beads are omitted. Color scheme: orange: dehydrophenylalanine; blue: phenylalanine; white: 2,6-naphthalenedicarboxyl.

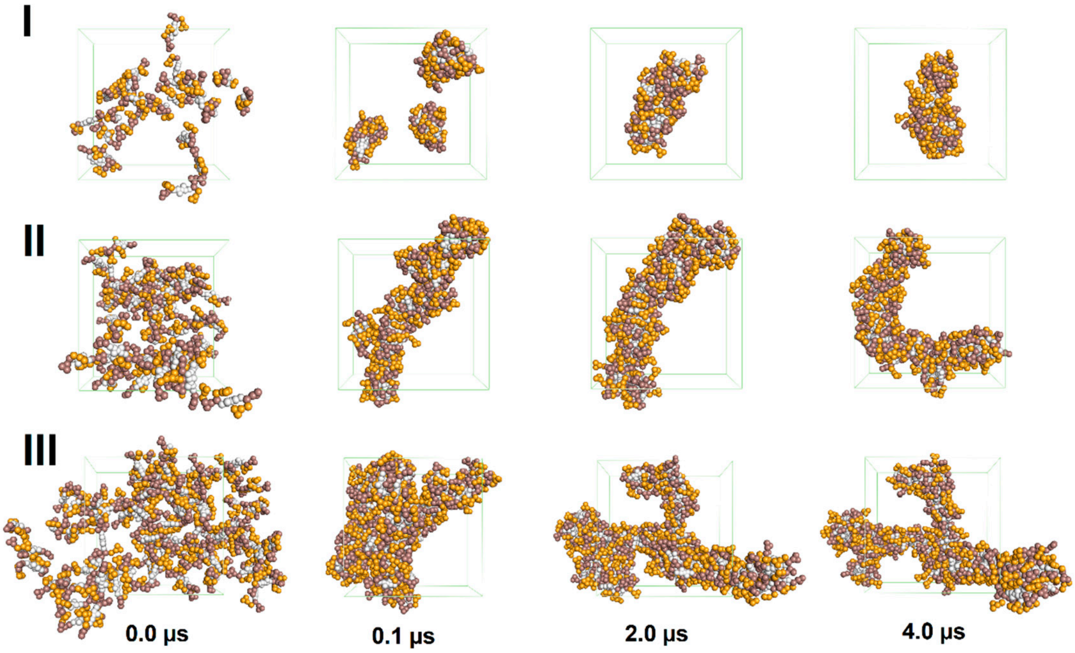

Figure S3. Snapshot at different time points of the self-assembly of compound **4** over a 4 μs long CG MD simulation in different concentrations: (I) 0.08 M; (II) 0.16 M; (III) 0.32 M. Water beads are omitted. Color scheme: orange: dehydrophenylalanine; brown: tyrosine; white: 2,6-naphthalenedicarboxyl.

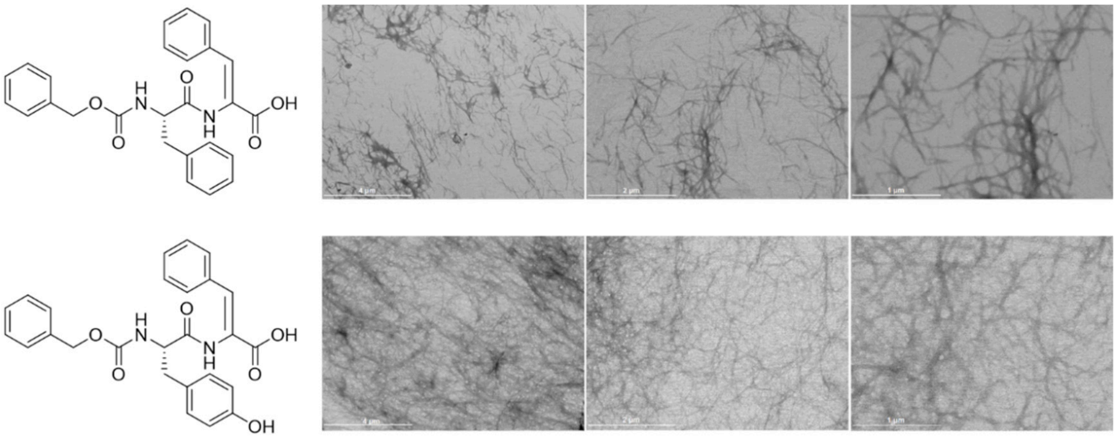

Figure S4. Scanning transmission electron microscopy (STEM) images of Cbz-Phe-ΔPhe-OH e Cbz-Tyr-ΔPhe-OH at 0.3 wt%, at different magnifications (4, 2 and 1 μm) [17].

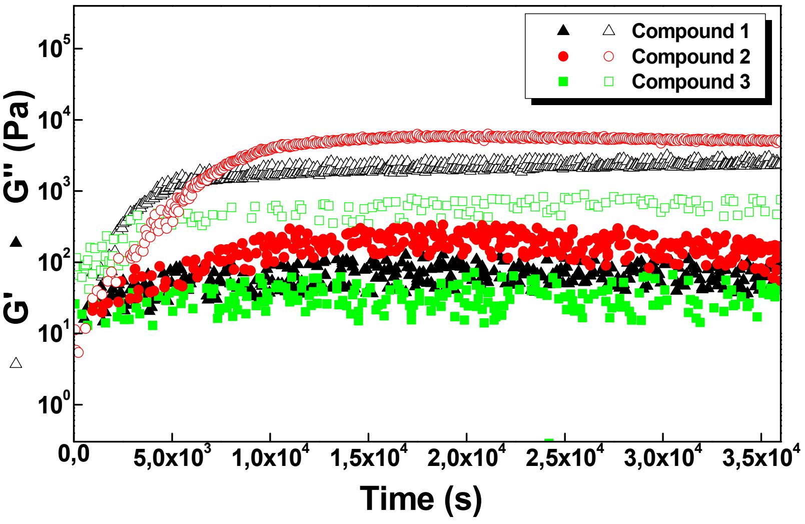

Figure S5. Elastic and viscous modulus during the kinetic process of gelation of compound **1**, **2** and **3**.

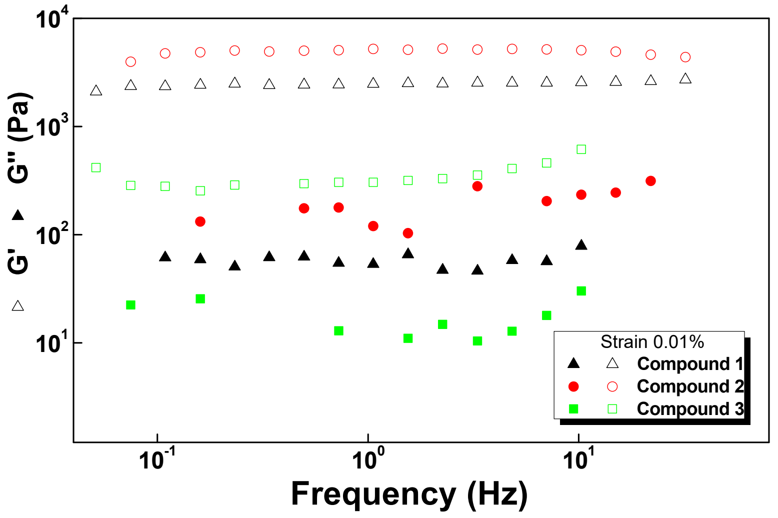

Figure S6. Frequency dependence of the shear elastic G′ (empty symbols) and loss G′′ (filled symbols) moduli for the compounds **1**, **2** and **3**.

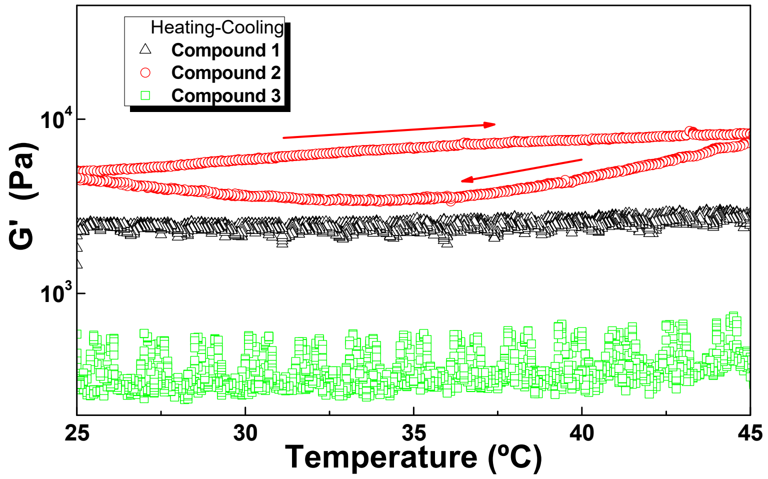

Figure S7. Thermal variation of the storage G′ recorded at a frequency of 1 Hz with a strain amplitude of 0.005%, during a heating and cooling cycle performed at a rate of 1 °C/min.

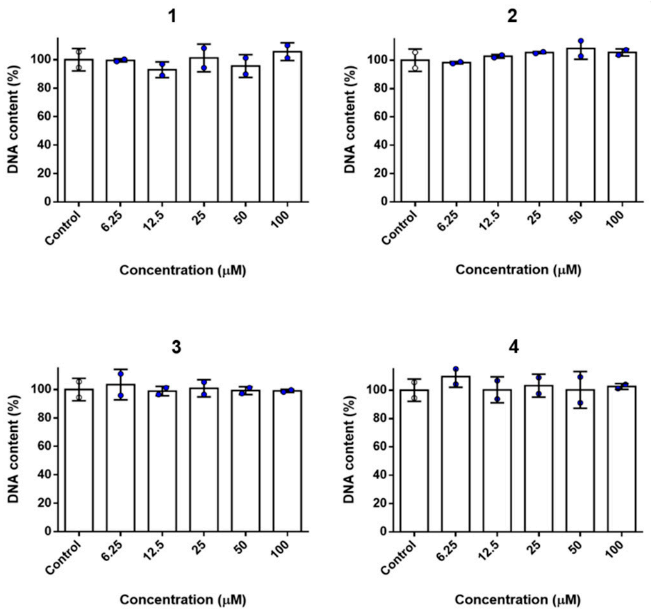

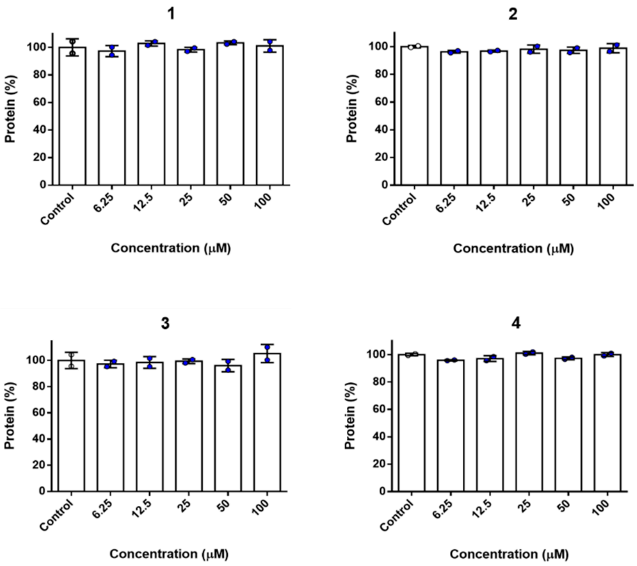

Figure S8. DNA and protein content of HaCaT cells in the same conditions as Figure 8.

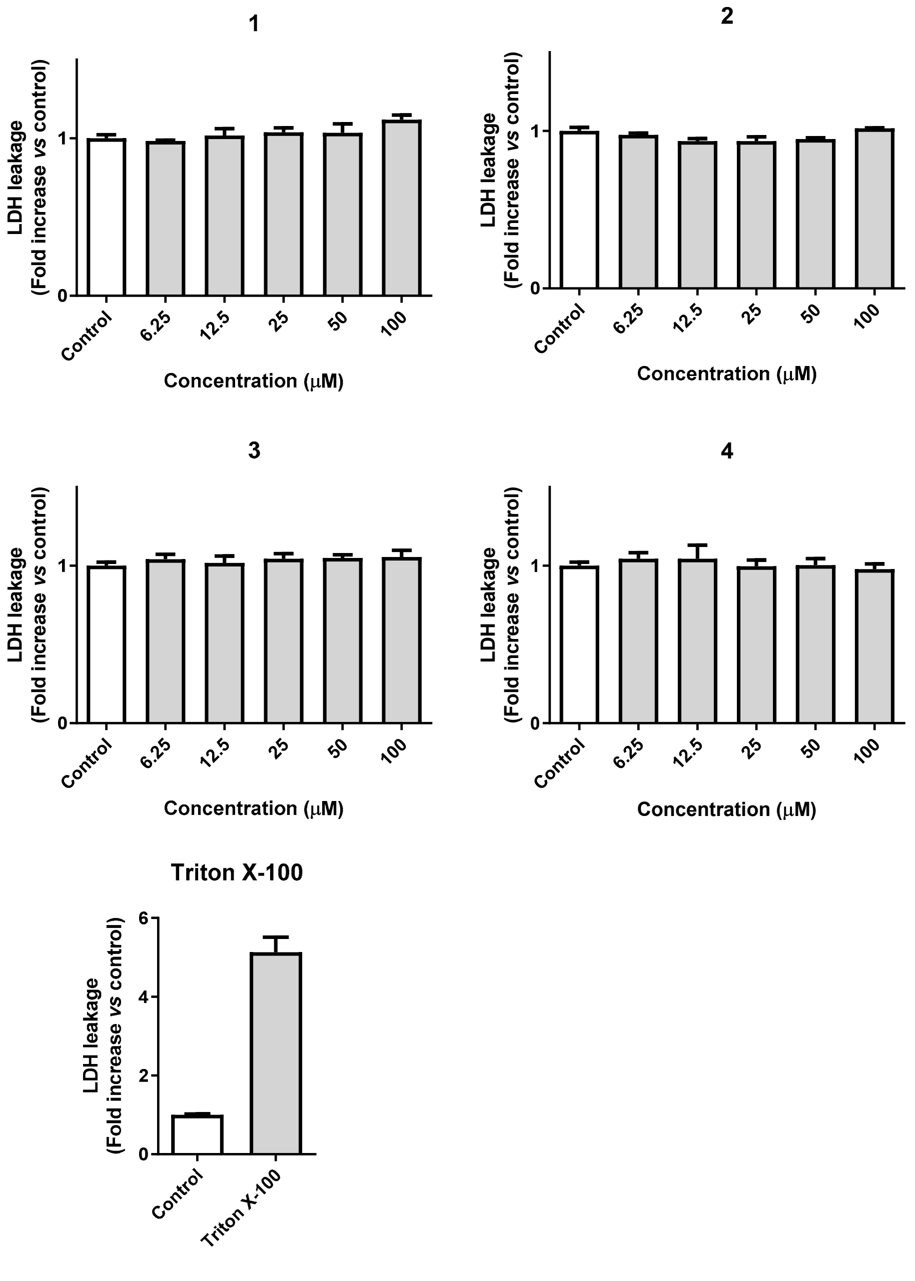

Figure S9. LDH activity found in the culture media of HaCaT cells treated with **1-4** for 24 h, at the concentrations presented. Triton X-100 was used as positive control to lyse cells.

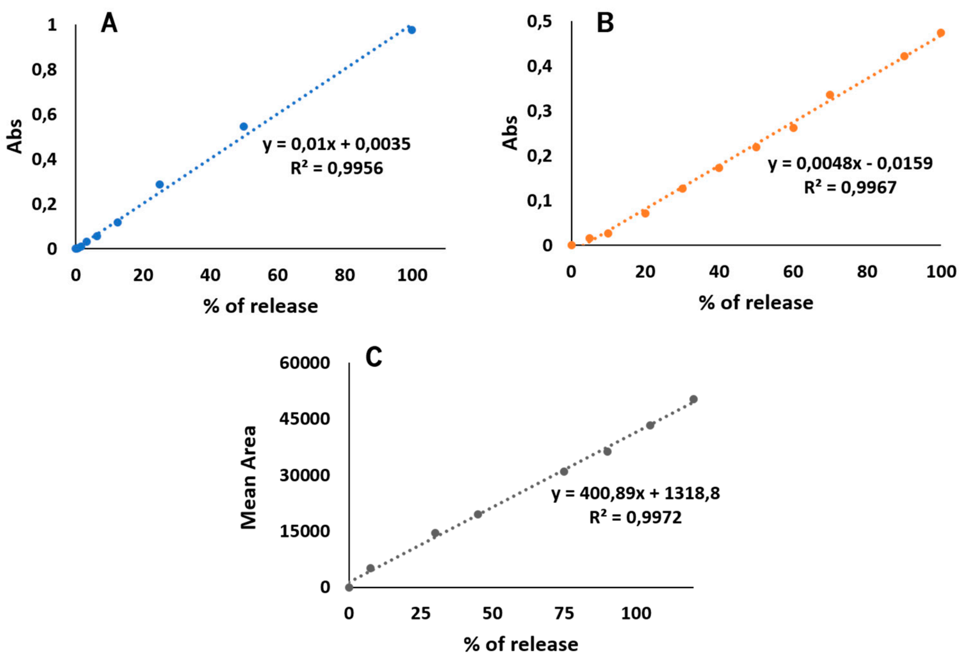

Figure S10 Calibration curve to determine the amount of cargo in present in the layered solution above the hydrogel. (**A**) Methylene blue was measured by UV-Vis spectroscopy by absorbance at 666 nm. Equation of linear correlation: y=0.01x + 0.0035. (**B**) Methyl orange was measured by UV-Vis spectroscopy by absorbance at 465 nm. Equation of linear correlation: y=0.0048x − 0.0159. (**C**) Ciprofloxacin was measured by HPLC by area under the curve of the ciprofloxacin peak. Equation of linear correlation: y=400.89x + 1318.8.

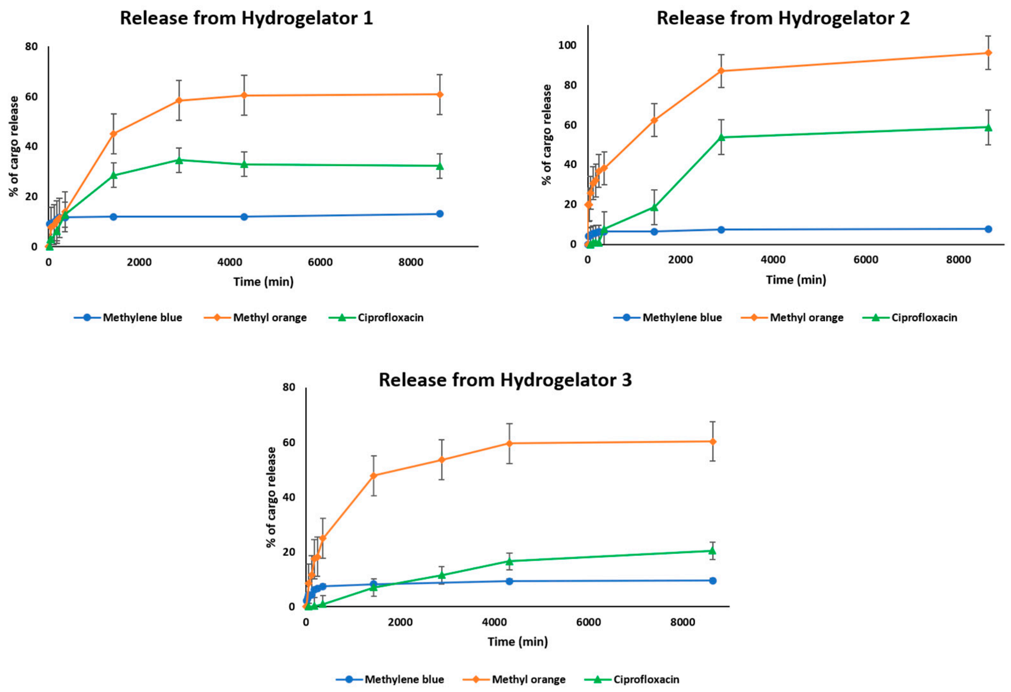

Figure S11. Percentage of cargo release vs time over 6 days. Release of methylene blue, methyl orange and ciprofloxacin from hydrogels of **1**, **2** and **3**.

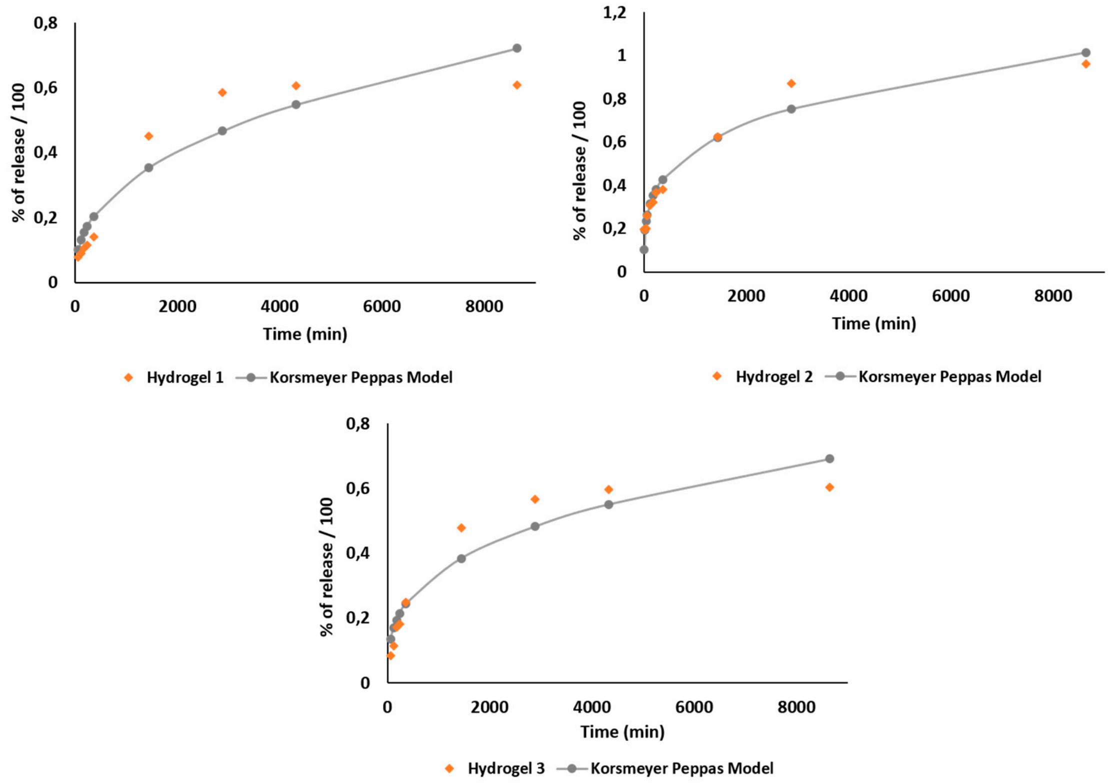

Figure S12. Data to Korsmeyer-Peppas Model to describe the release kinetics of methyl orange from hydrogels **1**, **2** and **3**.

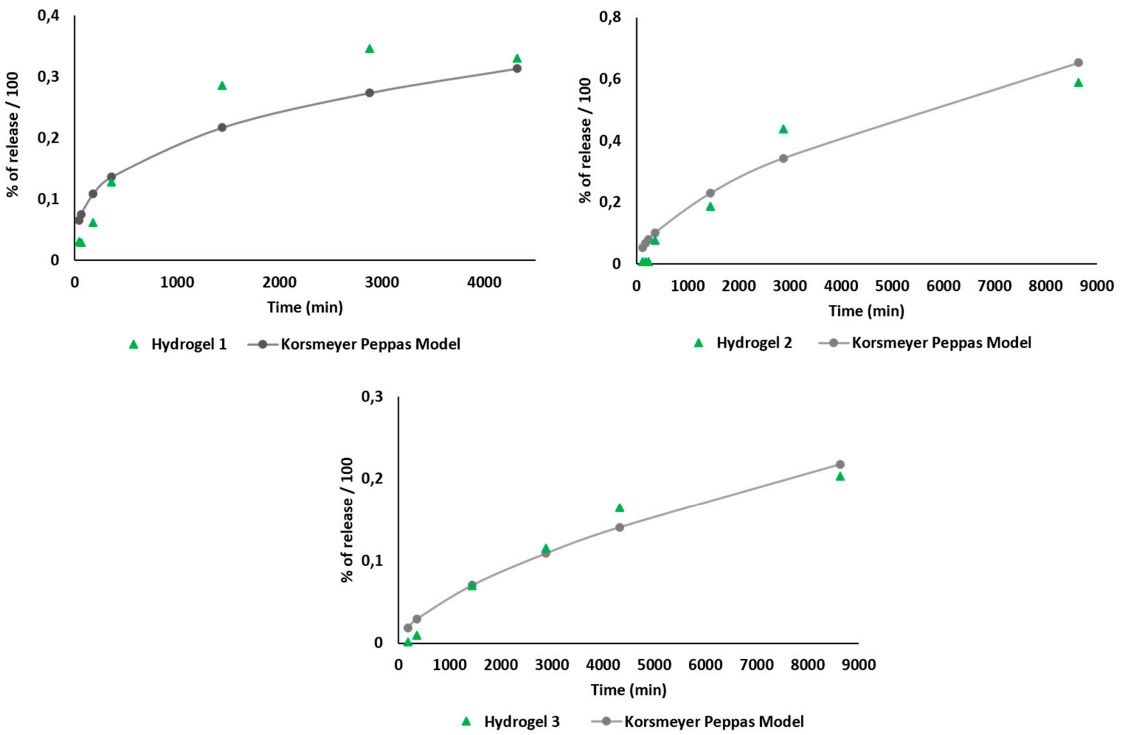

Figure S13. Data to Korsmeyer-Peppas Model to describe the release kinetics of ciprofloxacin from hydrogels **1**, **2** and **3**.

Table S1. Aggregation propensity score (AP) values obtained for the various simulations of compound **3** and **4**, and final SASA fraction of the dehydrophenylalanine (ΔPhe), tyrosine (Tyr), phenylalanine (Phe) and 2,6-naphthalenedicarboxyl (Naph) groups.

	**3**				**4**			
	**AP**	**Naph**	**Phe**	**ΔPhe**	**AP**	**Naph**	**Tyr**	**ΔPhe**
**0.08 M**	3.92	0.28	0.33	0.39	3.72	0.26	0.36	0.38
**0.16 M**	4.54	0.25	0.35	0.40	4.04	0.22	0.35	0.43
**0.32 M**	3.28	0.22	0.35	0.43	3.15	0.21	0.34	0.45
